# Reconstruction of pathway modification induced by nicotinamide using multi-omic network analyses in triple negative breast cancer

**DOI:** 10.1038/s41598-017-03322-7

**Published:** 2017-06-14

**Authors:** Ji Young Kim, Hyebin Lee, Jongmin Woo, Wang Yue, Kwangsoo Kim, Seongmin Choi, Ja-June Jang, Youngsoo Kim, In Ae Park, Dohyun Han, Han Suk Ryu

**Affiliations:** 1 Department of Pathology, Seoul National University Hospital, Seoul National University College of Medicine, Seoul, South Korea; 2 Department of Radiation Oncology, Kangbuk Samsung Hospital, Sungkyunkwan University School of Medicine, Seoul, South Korea; 3 Department of Biomedical Sciences, Seoul National University College of Medicine, Seoul, South Korea; 4 Division of Clinical Bioinformatics, Biomedical Research Institute, Seoul National University Hospital, Seoul, South Korea; 5 Proteomics Core Facility, Biomedical Research Institute, Seoul National University Hospital, Seoul, South Korea

## Abstract

Triple negative breast cancer (TNBC) is characterized by an aggressive biological behavior in the absence of a specific target agent. Nicotinamide has recently been proven to be a novel therapeutic agent for skin tumors in an ONTRAC trial. We performed combinatory transcriptomic and in-depth proteomic analyses to characterize the network of molecular interactions in TNBC cells treated with nicotinamide. The multi-omic profiles revealed that nicotinamide drives significant functional alterations related to major cellular pathways, including the cell cycle, DNA replication, apoptosis and DNA damage repair. We further elaborated the global interaction networks of molecular events via nicotinamide-inducible expression changes at the mRNA and functional protein levels. This approach indicated that nicotinamide treatment rewires interaction networks toward dysfunction in DNA damage repair and away from a pro-growth state in TNBC. To our knowledge, the high-resolution network interactions identified in the present study provide the first evidence to comprehensively support the hypothesis of nicotinamide as a novel therapeutic agent in TNBC.

## Introduction

Triple negative breast cancer (TNBC) accounts for 10–20% of all breast cancers and is generally associated with a worse prognosis than that of non-TNBC as a result of its aggressive biological behavior and the lack of targeted therapeutic strategies. A systemic chemotherapeutic regimen is the only reliable treatment option; however, pathologically complete remission occurs in approximately only one-third of cases^1^, which thus necessitates new drug development or the application of agents previously used for the treatment of other diseases, such as metformin^2^.

Nicotinamide is the active amide form of vitamin B3 or niacin, a precursor for the synthesis of nicotinamide adenine dinucleotide (NAD+). NAD has been implicated in genomic stability, apoptosis, cell signaling, stress tolerance, and metabolism^3^. A recent ONTRAC clinical trial has demonstrated the chemo-preventive effect of nicotinamide in superficial basal-cell carcinomas^4^. In a previous study, we also identified an inhibitory effect of nicotinamide on the development of pre-neoplastic lesions and the progression of differentiated HCC to a high-grade tumor in animal models^5^. However, most previous studies have reflected single or a limited number of molecular events, which thereby limits the current understanding of the entire spectrum of complex molecular alterations and interaction networks accompanied by nicotinamide treatment in cancer cells.

Interest in personalized cancer therapy has led to numerous advances in the field of cancer genomics, and next-generation sequencing represents one development that elaborates various types of genomic alterations. Nevertheless, it remains unclear whether genomic events are eventually translated into proteomes, which are the final functional units within a cell. In addition, previous studies have exhibited considerable discordance between genomic mutations and protein levels^6, 7^.

The rapid advances of proteomic technologies with computational algorithms and biochemical technologies have enabled quantitative analyses to evaluate the functional interaction networks, which reflect final cellular functions^8^; thus, this approach overcomes the limitations of genomic data that exhibit a low correlation between genomic mutations and the expression profiles of their corresponding peptides.

In the present study, we employed whole-transcriptome and global quantitative proteomic methods to characterize how genes and proteins are differentially expressed in nicotinamide-treated TNBC cells. In addition, the identification of the rewiring interaction networks associated with therapy provides additional evidence for the application of nicotinamide to enhance therapeutic benefits in TNBC.

## Results

### Nicotinamide inhibits TNBC cell growth

**Figure 1:**
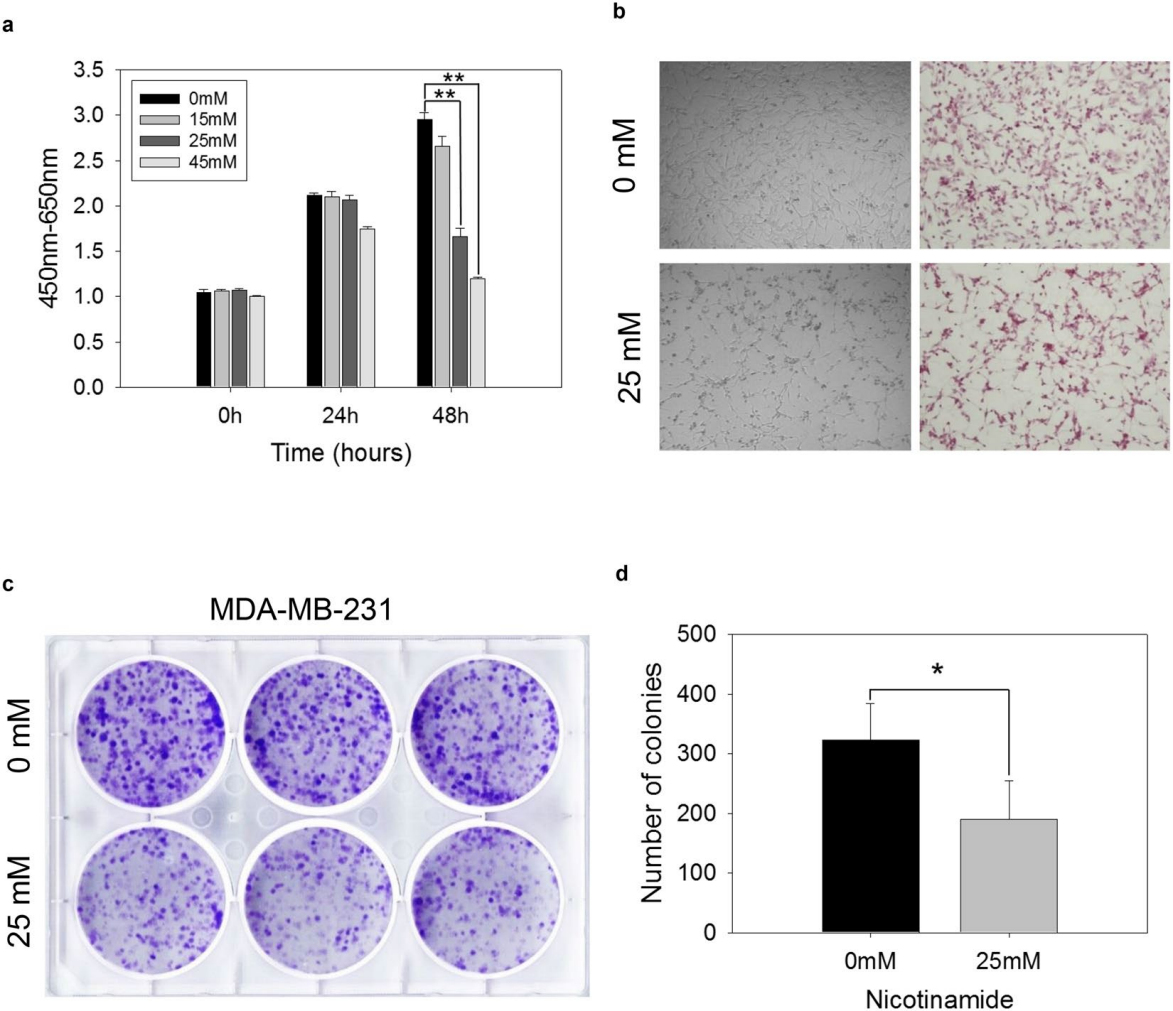
Effects of nicotinamide on breast cancer cell lines. (**a**) MDA-MB-231 cells were treated with 15, 25 and 45 mM nicotinamide. Dose-dependent cytotoxic effects were assessed every 24 and 48 h using a WST-1 assay. The data represent the mean ± SD (**P < 0.01). The cell morphology of MDA-MB-231 cells treated with 25 mM nicotinamide for 48 h via (**b**) light microscopy (left, original magnification x 100) and H&E staining (right, original magnification x 100). (**c**) Colony formation assays of MDA-MB-231 cells treated with nicotinamide 25 mM for 48 h. (d) The bar graph indicates colony numbers revealed by the colony formation assay. The data represent the mean ± SD (*P < 0.05).

To examine how nicotinamide affects cell growth, we treated three types of TNBC cells with nicotinamide in a dose-dependent manner and measured the cell viability using a WST-1 cell proliferation assay to identify the maximum tolerable dose of nicotinamide; the data indicated 25 mM to be the optimal dose of nicotinamide (Fig. 1a and Supplementary Fig. S1a). The results are consistent with suppression of growth in treated TNBC cells and support the loss of cell viability as documented in the WST-1 assay (Fig. 1b and Supplementary Fig. S1b﻿). A colony formation assay was performed to investigate the inhibitory function of nicotinamide on the cell viability, which indicated that tumor cells treated with nicotinamide exhibited significantly decreased colony numbers (Fig. 1c and d). Cell cycle analysis and apoptosis assay revealed that nicotinamide triggered S or G1 phase arrest and apoptosis in all kinds of TNBC cells, respectively (Supplementary Fig. S2). As a result, we confirmed an inhibitory effect of nicotinamide on cell growth in highly aggressive TNBC cells.

### Nicotinamide inhibits SIRT1 deacetylase activity

We further attempted to identify the primary target molecules of nicotinamide in TNBC cells. Previous studies have shown that nicotinamide inhibits SIRT1 enzymatic activity^9^. In the nicotinamide-treated cells, there was no significant difference in the SIRT1 protein expression. However, western blot analysis indicated that the acetylated-p53 protein expression was significantly increased (Supplementary Fig. S3). The acetylation of p53 represents an active form of p53, which is controlled by SIRT1^10^.

### In-depth analysis of transcriptome and proteomics

**Figure 2:**
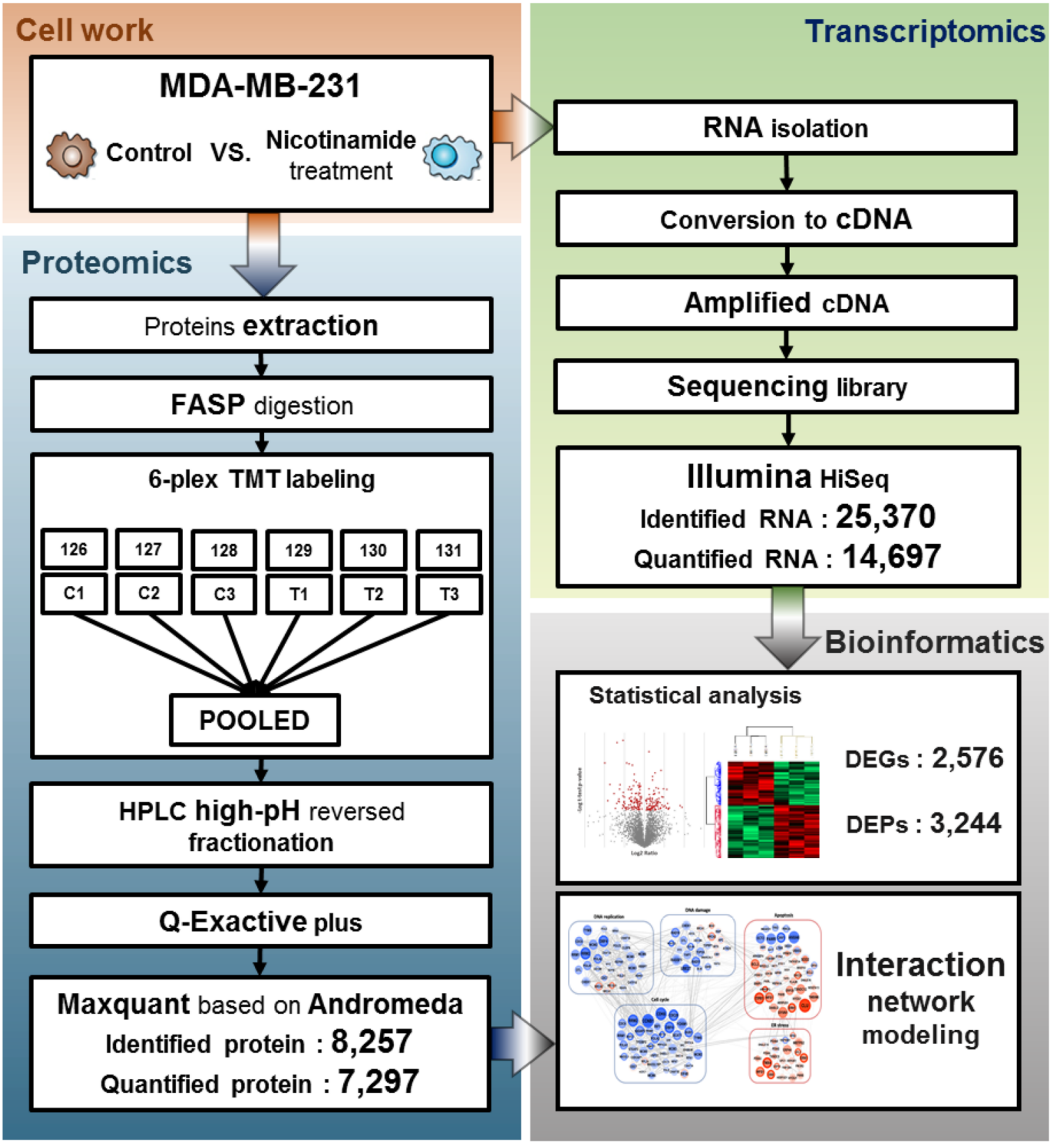
Schematic representation of the experiment workflow to analyze the changes in the transcriptome and proteome of MDA-MB-231 cells in response to nicotinamide.

To obtain a comprehensive perspective of the changes induced by nicotinamide, we determined the transcriptome and proteome profiles of MDA-MB-231 cells treated for 48 h with nicotinamide and untreated as a control (Fig. 2). The mRNA levels in the three biological replicates (Pearson’s correlations ranged from 0.929 to 0.993) were determined using RNA-sequencing, which enabled the quantification of 14,697 transcripts (Supplementary Table S1 and Supplementary Fig. S4a). To measure the protein expression levels, we compared untreated and nicotinamide-treated MDA-MB-231 cells in biological triplicate with mass spectrometry-based protein quantitation using TMT isobaric tagging. In total, we identified 93,978 unique peptides that corresponded to 7,297 protein groups quantified across all six samples (Supplementary Table S2 and Supplementary Fig. S4b). In regard to the reproducibility of the three biological replicates, we determined that the Pearson’s correlation for each pair of replicates was >0.99 (Supplementary Fig. S4). The median values for the coefficient of variation (CV) for the abundance of proteins between the biological replicates were 2.8% and 1.4% in the non-treated and treated conditions, respectively (Supplementary Fig. S5).

To quantify the changes in the transcriptome and proteome, we set the statistical significance level at *P* value < 0.05 with minimal fold-changes of ± 2 (transcriptome) and ± 1.2 (proteome). We identified 1,447 genes of higher abundance when MDA-MB-231 cells were treated with nicotinamide, whereas 1,129 genes were of lower mRNA abundance (Supplementary Table S3). In the proteome, a comparative analysis between the non-treated and treated cells identified 3,244 differentially expressed proteins (Supplementary Table S4).

We subsequently compared the transcriptome and proteome changes in response to nicotinamide. For 90% of the proteins quantified by MS, we also obtained an estimate of the mRNA abundance using RNA-sequencing. The Pearson correlation of the transcript and protein abundance is approximately 0.45, which is consistent with previous studies^11^. We also analyzed the correlation of nicotinamide-induced changes at the transcript level with changes at the proteome level, and the results exhibited a similar correlation (Pearson’s correlation 0.56) consistent with previous findings^11^. The correlation between the proteomes and corresponding transcriptomes was only 0.4 to 0.5; thus, these results indicated important differences between the transcriptome and proteome^11, 12^.

### Global pathway regulation in response to nicotinamide in breast cancer cells

**Figure 3:**
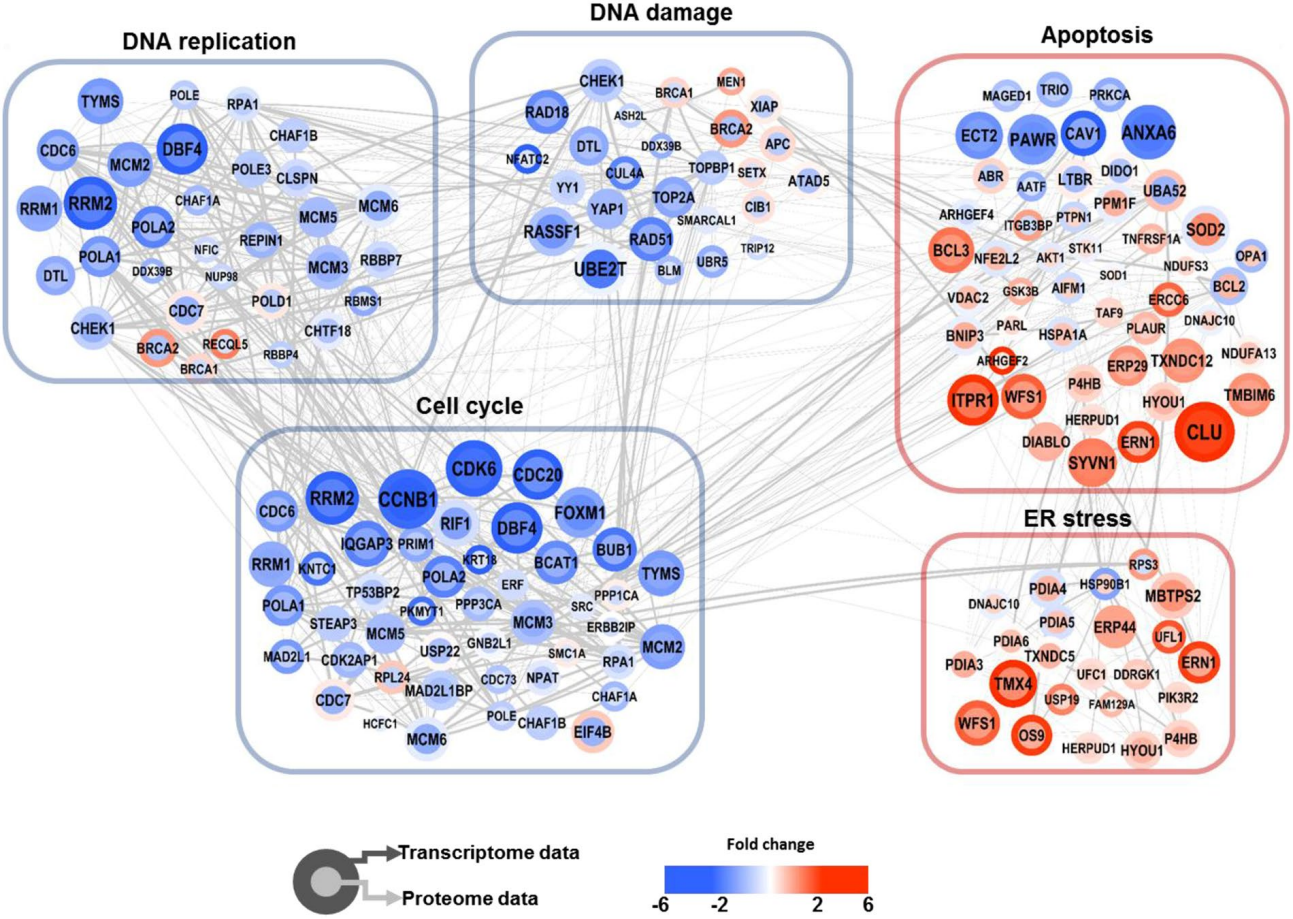
Overview of the interaction network model. The overall organization of the protein-protein interaction was visualized using Cytoscape 3.4.0. Colors of the border and inside nodes indicate the expression levels of quantified proteins and mRNAs, respectively. Gray edge line thickness defines the protein-protein interaction score identified in the STRING public database. Blue and pink boxes are clustered proteins, which are significantly enriched gene ontology biological process terms derived from the DAVID functional annotation classification tool with *P*-values < 0.05 classified as significant using a modified Fisher’s exact test.

To characterize the global effect of nicotinamide in breast cancer cells on the protein and transcript levels, we performed a gene ontology term and KEGG enrichment (Supplementary Tables S5 and S6 and Supplementary Fig. S6) and network analysis. Interestingly, we observed that the cell cycle, DNA replication and DNA damage responses were significantly down-regulated at both the transcriptome and proteome levels (Fig. 3).

In addition, we identified a significant up-regulation of many proteins associated with apoptosis and the ER-stress response at the transcriptome and proteome levels, which is consistent with the suppression of cell growth in nicotinamide-treated cells^5^. However, we found that the pathways involved in the metabolic process were up-regulated at both the transcriptome and proteome levels in the nicotinamide-treated cells. Previous studies have shown that nicotinamide boosts energy reservoirs in the mitochondria^13^. These studies suggested that nicotinamide treatment may up-regulate metabolic processes in MDA-MB-231 cells.

To exploit a global view of proteins that act together in nicotinamide-treated cells, we constructed functional networks based on gene ontology and pathway enrichment. This analysis indicated links between distinct cellular processes in nicotinamide treated cells. Previous studies have shown the anti-proliferative effect of nicotinamide; however, the underlying mechanisms have not been described in detail. Thus, the present study showed an interaction network related to the anti-proliferative effect of nicotinamide in MDA-MB-231 cells at the transcriptome and proteome levels.

### Nicotinamide induced cell cycle arrest

**Figure 4:**
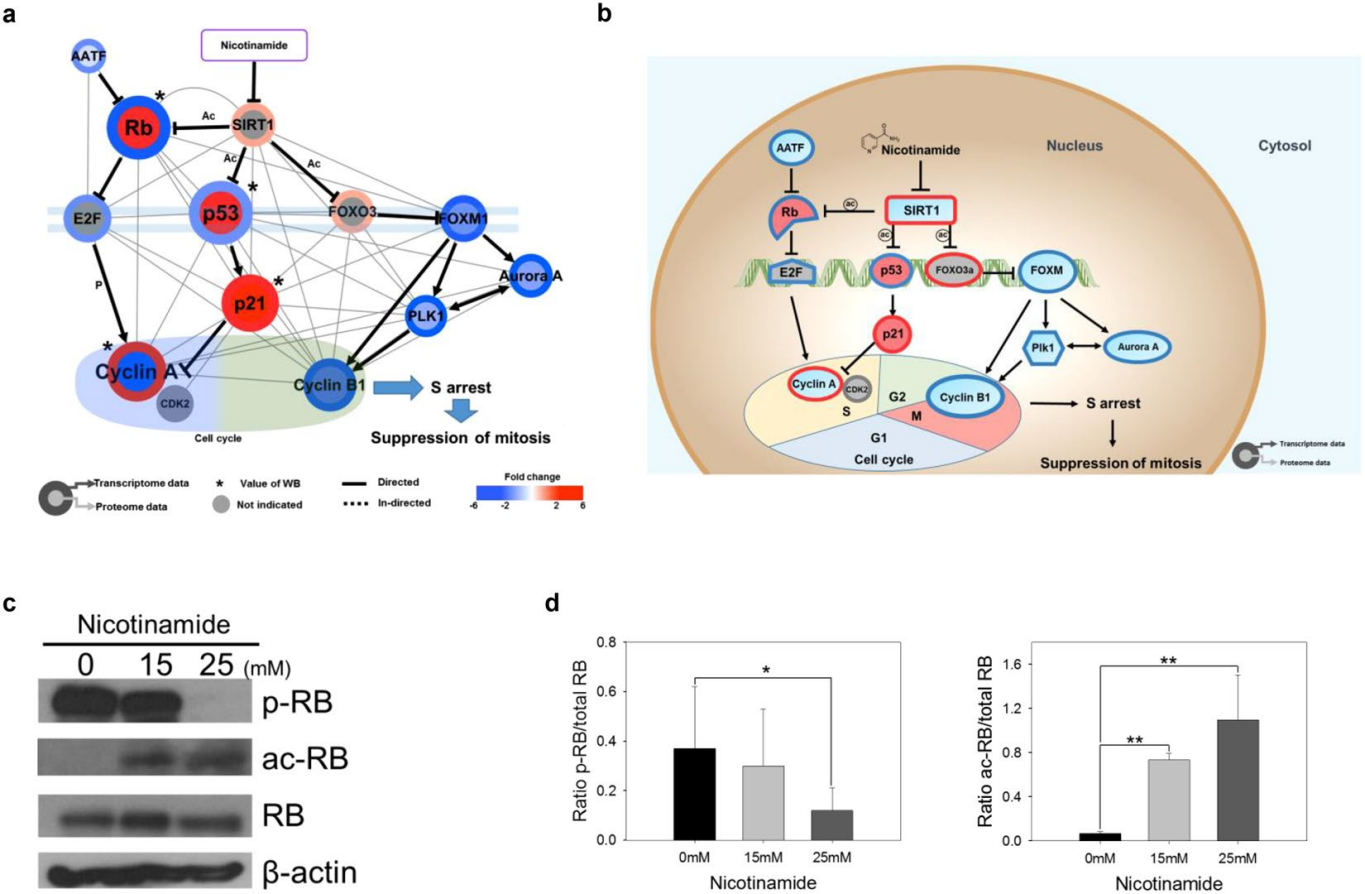
Nicotinamide induces cell cycle arrest. (**a**) Cytoscape visualization of cell cycle regulatory network. The colors of the border and inside circle indicate the expression levels of transcriptome and proteome data, respectively. Gray edge indicates the protein-protein interaction based on the STRING database, whereas the arrows and inhibition symbols represent the activation and repression information, respectively. Nodes with asterisks indicate that the protein expression was assessed by only western blotting without the use of proteome data. There is no significant difference regarding the expression level if the color of the node is gray. (**b**) Proposed model of nicotinamide-induced cell cycle interaction network. Networks are represented by proteome, transcriptome and western blotting data. Red indicates increased expression, blue indicates decreased expression and gray indicates no significant difference. (**c**) Expression of RB and the phosphorylation and acetylation of RB in nicotinamide-treated cells were examined via western blotting. (**d**) Densitometry analysis of p-RB and ac-RB was quantitated using ImageJ. Values were normalized to the levels of RB. The data represent the mean ± SD (*P < 0.05, **P < 0.01). Abbreviations: ac - acetylation, p - phosphorylation, WB - western blot.

There is accumulating evidence for the regulation of the cell cycle by SIRT1, which primarily promotes cell cycle progression^14^. Quantitative proteomics demonstrated that the altered expression of 47 peptides modulates the cell cycle. Among their proteomic signatures, 40 protein expression profiles overlapped with mRNA expression, and most of the proteins showed concomitantly reduced mRNA and protein expression according to the Cytoscape analysis (38/47, 80.8%, Fig. 3). In searches using multiple public data resources from the integration of multi-omic expression profiles and information extraction using a literature mining approach, selected 13 factors were predominately involved in the S and G2/M phases (Fig. 4a and b). Initially, these findings prompted the determination of which phase would be arrested during the cell cycle; thus, we performed FACS and confirmed that nicotinamide treatment primarily impeded the cell cycle through the S phase (Supplementary Fig. S2). In the subsequent step, to characterize how cell cycle progression interaction networks are rewired after nicotinamide treatment, we adopted a strategy based on the known interaction networks from systematic text-mining mapped through nicotinamide-induced pathway reconstruction in the transcriptome and proteome profiles obtained in the present study.

The first observation was a substantial decrease of cyclin A (CCNA) expression which was simultaneously measured via western blot analysis. There was no significant difference in mRNA levels of cyclin A (CCNA) (Supplementary Fig. S7). In additional interaction network analyses that utilized bioinformatics pathway/network evaluations, two main streams, including 1) RB-E2F and 2) p53–p21 (TP53-CDKN1A) axis, which ultimately down-regulate cyclin A (CCNA) expression, were identified, which in turn induced cell cycle arrest at the S phase (Fig. 4a and b). Cyclin A (CCNA) is regulated by E2F, which is previously inactivated by the binding of dephosphorylated- or acetylated-RB upstream^15^. According to previous studies, top-down commands from nicotinamide impair SIRT1 activity, which in turn revitalizes RB activity through acetylation^16^ and results in the inactivation of E2F, a crucial transcription factor for cyclin A (CCNA)^17^. These findings are consistent with the increased dephosphorylated- or acetylated-RB protein expression identified using western blot following the ablation of SIRT1 enzymatic activity induced by nicotinamide treatment (Fig. 4c and d).

In addition to the RB and E2F complex axis, in the present study, the mRNA and protein expression of p21 (CDKN1A) was significantly increased in the combinatory transcriptomic and proteomic analyses of the acetylation of p53 (TP53) protein after nicotinamide administration (Supplementary Fig. S7). As a cell-cycle checkpoint, p21 (CDKN1A), a pivotal downstream effector of p53 (TP53), mediates the inactivation of various cyclin-CDK complexes^17^. Previous studies have also revealed that reduced SIRT1 activity eventually triggered p21 (CDKN1A) signaling activity through the resurgent p53–p21 (TP53-CDKN1A) signaling pathway^18^.

In the present study, we also identified the rewiring of two novel molecular signals: 1) cyclin B1 (CCNB1), polo-like kinase (PLK1), Aurora A (AURKA) and 2) anaphase-promoting complex/cyclosome (APC/C, ANAPC)-CDH1 complex, mainly engaged in G2/M phase progression. The first three factors are controlled by a transcription factor referred to as FOXM1^19^. In the present study, the FOXM1 and FOXO3 expression was significantly decreased or increased either at the mRNA or protein levels, respectively (Fig. 4a and Supplementary Fig. S7). FOXM1 is inhibited by FOXO3^20^, which is controlled by SIRT1^21^. The APC/C (ANAPC)-CDH1 complex, another signaling axis related to the G2/M phase, primarily mediates the degradation of regulators of cytokinesis and centrosome replication and targets the A/B types of mitotic cyclins and FOXM1 for destruction^22^ for the negative regulation of Aurora A (AURKA) and PLK1^23^. These results also demonstrated that the expression of these factors was significantly decreased following nicotinamide treatment, which caused the reactivation of the APC/C (ANAPC)-CDH1 complex and played another role in G2/M arrest through cyclin B1 (CCNB1) inactivation. Taken together, these findings suggest that the treatment of nicotinamide provokes novel interaction networks and negatively impacts cell cycle progression through the G2/M border, where the agent initially exhibits a negative feedback to SIRT1, followed by sequential signaling modifications of FOXO3 and FOXM1, which eventually disrupt G2/M phase progression. In addition, the repression of Aurora A (AURKA), PLK1, and cyclin B1 (CCNB1) by the APC/C (ANAPC)-CDH1 complex terminates cell cycle progression at G2/M.

### Nicotinamide inhibits multiprotein complex interplay between DNA replication

**Figure 5:**
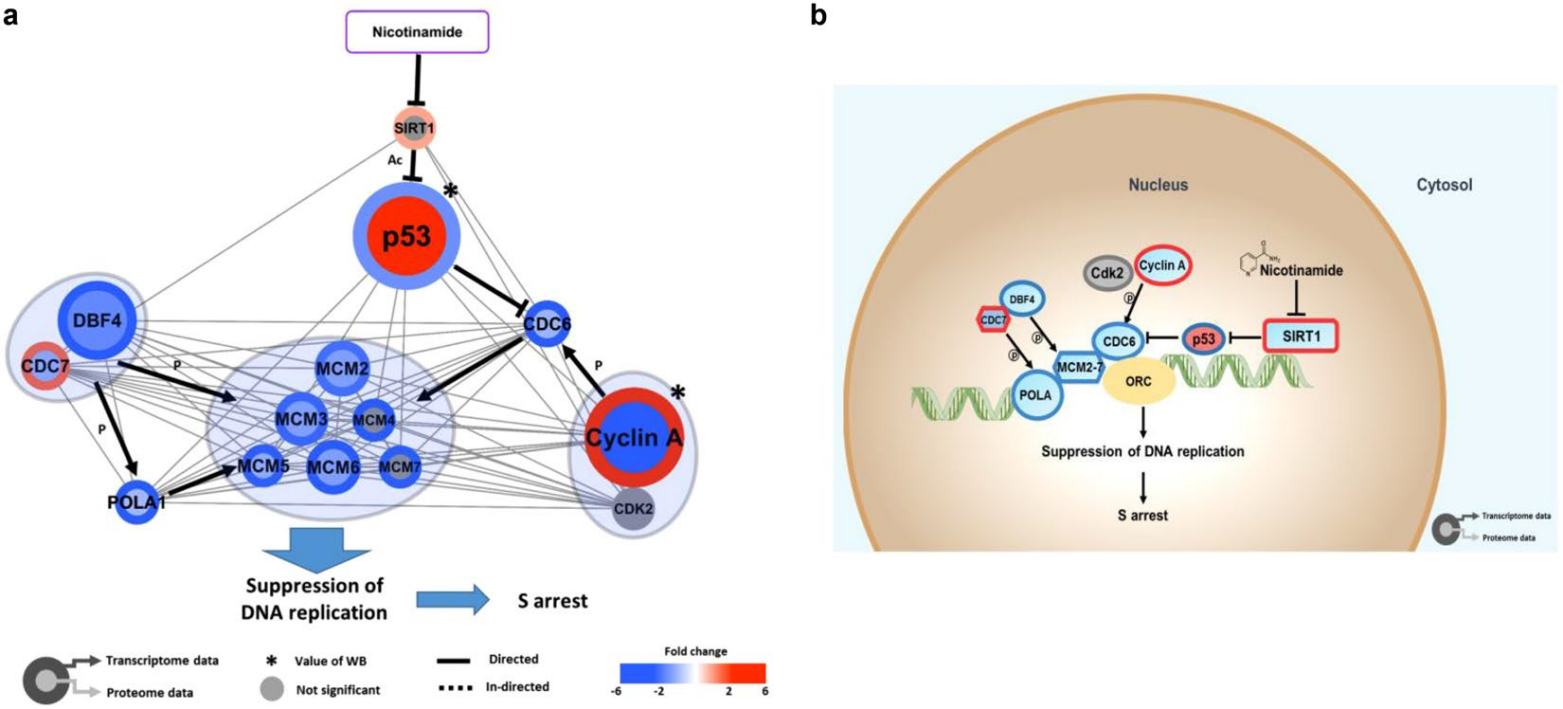
Regulatory network model of DNA replication coupled with nicotinamide. (**a**) The colors of the border and inside circle indicate the expression levels of transcriptome and proteome data, respectively. Gray edge indicates the protein-protein interaction based on the STRING database, whereas the arrows and inhibition symbols represent the activation and repression information. Nodes with asterisks indicate that the protein was assessed by only western blotting without the use of proteome data. There is no significant difference regarding the expression level if the color of the node is gray. Blue circle clustering highlights the complex enzymes of proteins. (**b**) Proposed model of nicotinamide-induced DNA replication interaction network. Networks are represented to reflect proteome, transcriptome and western blotting. Red indicates increased expression, blue indicates decreased expression and gray indicates no significant difference. Abbreviations: ac - acetylation, p - phosphorylation, WB - western blot.

In addition to a close interaction between nicotinamide and p53 (TP53) in cell cycle progression as previously described in the present study, this agent also exhibited induced changes in the expression of 33 DNA replication complex-related mRNAs and proteins (Fig. 3). Based on previous evidence, we further employed an interaction network analysis, including text-mining methods, to reconstruct a rewired molecular pathway. As a result, nicotinamide modified global transcriptomes and proteomes toward DNA replication complex dysfunction through the strong suppression of several components, such as CDC6, CDC7/DBF4, the mini-chromosome maintenance protein (MCM) complex and polymerase-alpha (POLA), which constituted a complex by which we formulated a more exquisite interaction network triggered by nicotinamide treatment (Fig. 5a and b). RT-PCR also validated reduced single gene’s expression including MCM2, 3, 6 and CDC6, which is coincide with our transcriptomes data (Supplementary Fig. S8). p53 (TP53) contributes to CDC6 inhibition^24^, which results in a decreased binding affinity to origin-recognition complexes (ORC), and the formation of the pre-replication complexes (pre-RC), followed by the recruitment of the DNA replicative helicase MCM complex^25^. In addition, the phosphorylation of POLA and MCM protein binding at pre-RC is decreased as a result of the decreased transcript or protein expression of the CDC7/DBF4 complex^26^, and these interaction flows reflect the impairment of normal DNA replication processes.

### Nicotinamide accelerates DNA damage in cancer cells via DNA repair dysfunction

Once DNA is damaged, the S phase checkpoint responds and further postpones the progression of the cell cycle and DNA replication. In the present study, we initially identified S phase arrest followed by nicotinamide treatment (Supplementary Fig. S2) thus, we hypothesized that nicotinamide-induced S phase arrest may reflect treatment-induced DNA damage.

**Figure 6:**
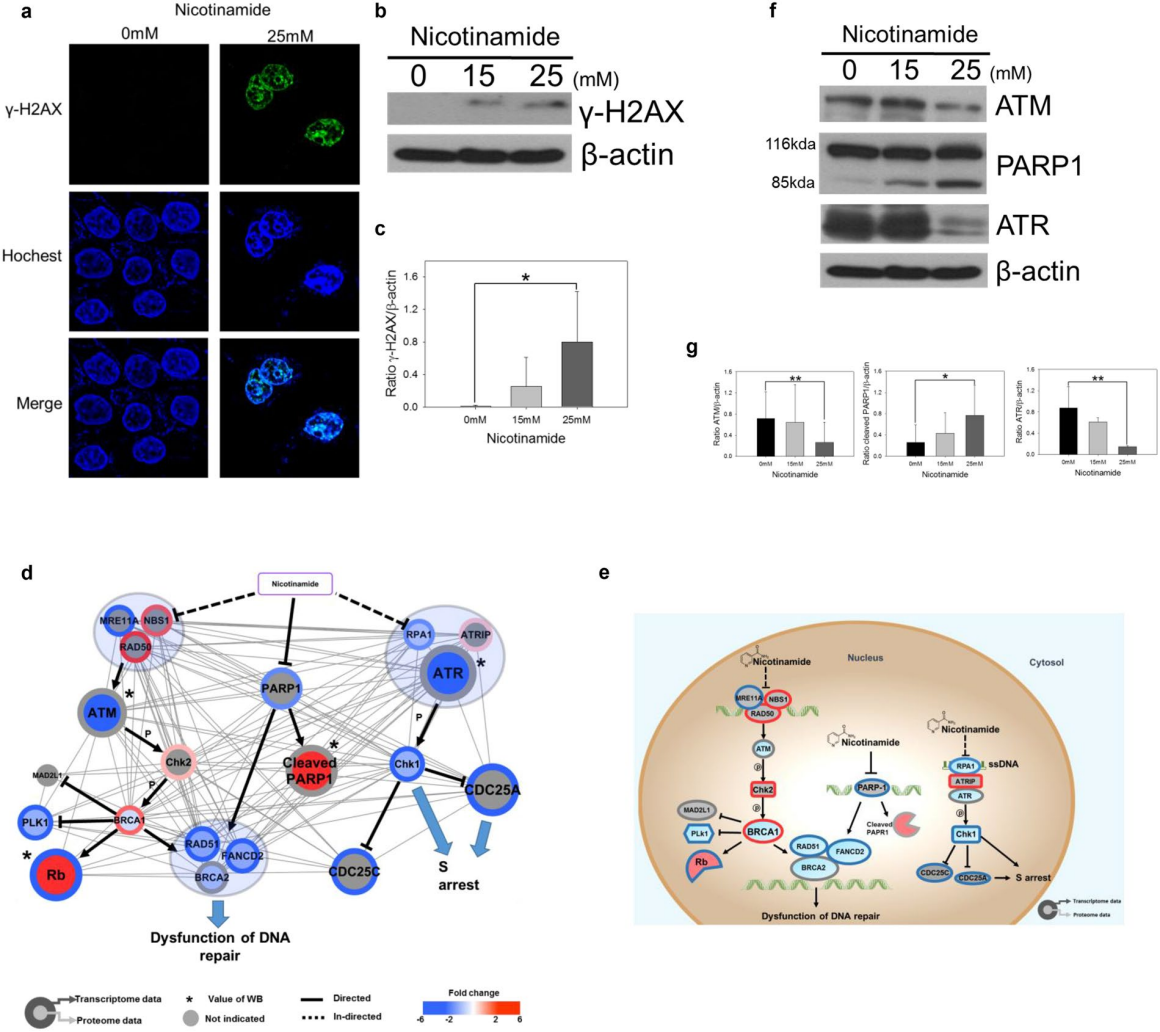
Nicotinamide suppresses DNA repair response. (**a**) Immunofluorescence staining of MDA-MB-231 cells incubated with 0 or 25 mM nicotinamide for 48 h. Green indicates γ-H2AX (H2AFX) foci, and blue indicates nuclei stained with Hoechst 33342. (**b**) Expression of γ-H2AX (H2AFX) in nicotinamide-treated cells were examined via western blotting. (**c**) Densitometry analysis of γ-H2AX (H2AFX) was quantitated using ImageJ. Values were normalized to the levels of β-actin. The data represent the mean ± SD (*P < 0.05). (**d**) Cytoscape visualization of cell cycle regulatory network. The colors of the border and inside circle indicate the expression levels of transcriptome and proteome data, respectively. Gray edge indicates the relationship of interacted/binding proteins based on the STRING database, whereas a strong solid and dotted line represent the relationships between proteins that are directly and indirectly affected, respectively. The arrows and inhibition symbols represent the activation and repression information. Node with asterisk mark indicates that the protein was assessed by only western blotting without the use of proteome data. There is no significant difference regarding the expression level if the color of the node is gray. Blue circle clustering highlights the complex enzymes of proteins. (**e**) Proposed model of nicotinamide-induced DNA repair interaction network. Networks are represented to reflect proteome, transcriptome and western blotting. Red indicates increased expression, blue indicates decreased expression and gray indicates no significant difference. (**f**) Expression of ATM, PARP1 and ATR in nicotinamide-treated cells was examined via western blotting. (**g**) Densitometry analysis of ATM, PARP1 and ATR was quantitated using ImageJ. Values were normalized to the levels of β-actin. Densitometric analysis was performed using ImageJ. The data represent the mean ± SD (*P < 0.05, **P < 0.01). Abbreviations: p - phosphorylation, WB - western blot.

To obtain insight into nicotinamide-induced DNA damage, we assessed γ-H2AX (H2AFX), an indicator of DNA damage, by immunofluorescence and western blotting; augmented γ-H2AX (H2AFX) expression was identified in the treated TNBC cells (Fig. 6a–c). These findings prompted us to evaluate more elaborate interaction networks rewired through the administration of nicotinamide; therefore, a network analysis was conducted based on multi-omics and western blotting combined with text mining. As indicated in Fig. 3, we identified a combinatory reduction of the expression of 19 mRNAs and proteins associated with DNA damage and repair among 28 signatures that exhibited a significant fold-change (67.9%). Consequently, three major pathways and related downstream interaction networks were reconstructed as 1) PARP1-related, 2) ATM-related and 3) ATR-related signaling pathways (Fig. 6d and e).

The cleaved form of PARP1 was significantly increased in the samples treated with nicotinamide as an inactive form of PARP1. Previous studies have also indicated nicotinamide as an exogenous inhibitor that impairs the proper function of PARP1, which primarily involves the repair of damaged DNA^27^. ATM recognizes double strand DNA breaks via the Mre11-Rad50-Nbs1 (MRE11-RAD50-NBN1, MRN) complex^28^.

In the present study, we used RT-PCR and western blot to confirm a significant decrease of ATM expression in the nicotinamide-treated cells (Fig. 6f and g and Supplementary Fig. S9) and transcriptomic changes of MRN expression, which forms a complex signaling axis with ATM by being placed in the upstream region.

Eventually, the complex activates ATM and induces the migration of the repair protein to sites of DNA double-strand breaks. The activation of downstream BRCA1, 2, RAD51 and FANCD2 regulated by the ATM-Chk2 (CHEK2) axis^29^ was also significantly decreased by nicotinamide treatment in our combinatory proteomics and transcriptomics which eventually hinder the formation of the DNA repair complex and the function of HR-mediated DNA repair. Also, transcript levels of BRCA2, RAD51 and FANCD2 were decreased in nicotinamide treated cells, which were consistent with transcriptomic analysis (Supplementary Fig. S9)

The expression of ATR, another marker of the DNA damage response and repair, was not altered in RT-PCR assays but significantly decreased in the blotting assay (Fig. 6f and g and Supplementary Fig. S9). In addition, the combined mRNA or protein expression of ATR-interacting protein (ATRIP), replication protein A (RPA) and Chk1 (CHEK1) located up- and downstream of ATR was significantly altered in our multi-omic analyses following the administration of nicotinamide (Fig. 6d and e). ATRIP, which is the regulatory partner of ATR, directly binds to RPA-ssDNA; thus, the ATR-ATRIP complex is localized to sites of DNA damage^30^. A preferred substrate of ATR is the signal transducing kinase Chk1 (CHEK1)^31^. Activated Chk1 (CHEK1) phosphorylates numerous substrates, including CDC25A and CDC25C, which are involved in cell-cycle progression at the S and G2/M transitions, respectively^32^. Our interaction network analysis indicates that several crucial players in the control of damaged DNA repair are globally dysregulated after nicotinamide treatment, which eventually impairs the restoration system of cancer cells from hazardous stimulations, such as chemotherapy.

### Nicotinamide induces apoptosis via p53 and endoplasmic reticulum stress

Our global interaction network indicated nicotinamide leads to the up-regulation of apoptosis-related networks and ER stress inducible signals, as well as a reduction in the cell cycle progression signal as previously described in the present study (Fig. 3). For further confirmation, we performed a flow cytometry-based Annexin-V/propidium iodide (PI) cell death assay in triplicate and observed nicotinamide induced the early and late stages of apoptosis (Supplementary Fig. S2), which reconfirmed the previous study^33^.

**Figure 7:**
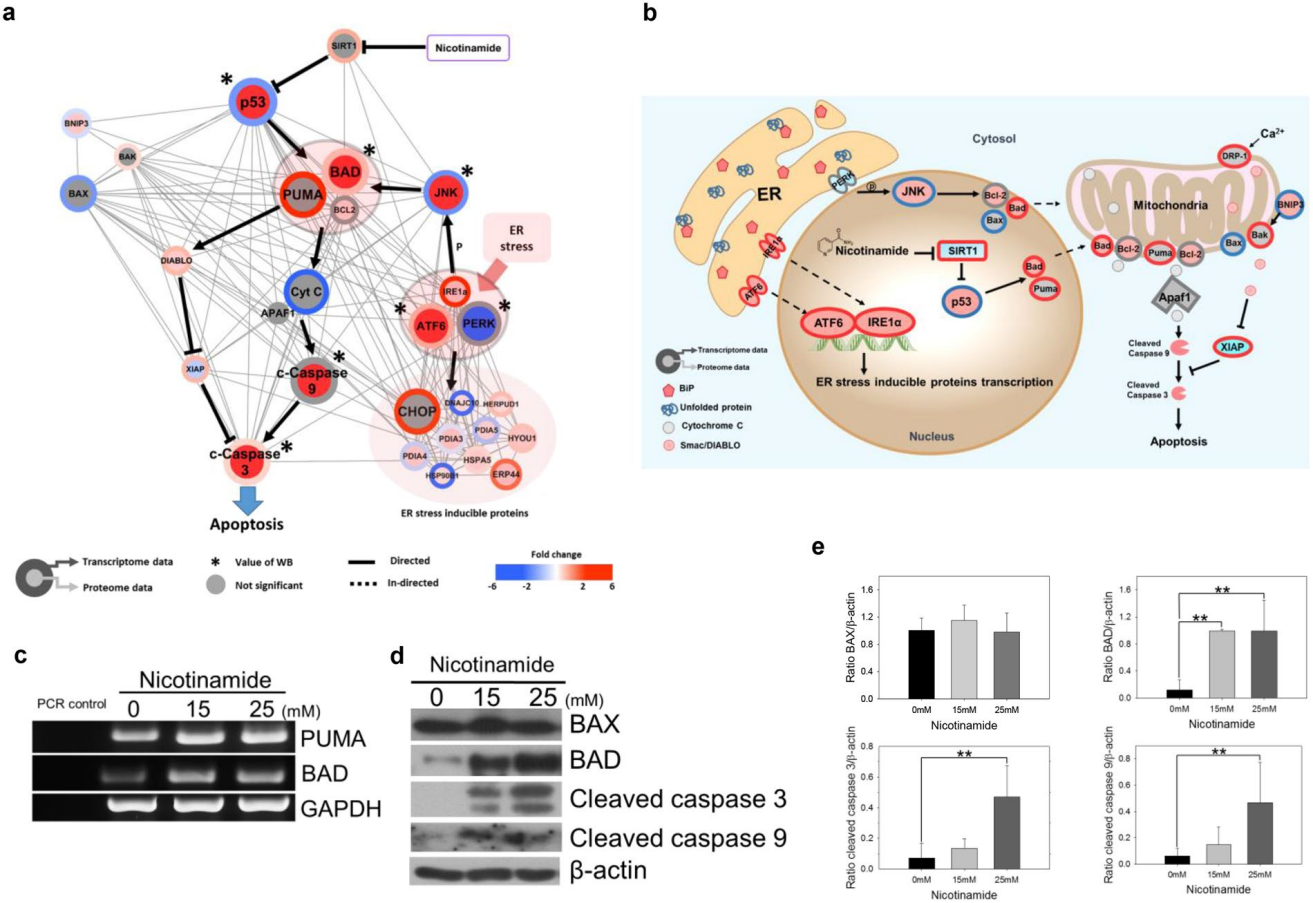
Nicotinamide induces apoptosis in MDA-MB-231 cells. (**a**) Cytoscape visualization of apoptosis regulatory network. The colors of the border and inside circle indicate the expression levels of transcriptome and proteome data, respectively. Gray edge indicates the protein-protein interaction based on the STRING database, whereas the arrows and inhibition symbols represent the activation and repression information, respectively. Nodes with asterisks indicate that the proteins were assessed by only western blotting without the use of proteome data. There is no significant difference regarding the expression level if the color of the node is gray. Pink circle clustering highlights the complex enzymes of proteins. (**b**) Proposed model of nicotinamide-induced apoptosis interaction network. Networks are represented to reflect proteome, transcriptome and western blotting. Red indicates increased expression, blue indicates decreased expression and gray indicates no significant difference. (**c**) The mRNA expression of PUMA (BBC3) and BAD was detected by RT-PCR. PCR control indicated negative control for RT-PCR mix. Expression of GAPDH was measured as a control for RNA integrity. (**d**) The apoptosis-related proteins BAX, BAD, cleaved caspase-9 (c-CASP9) and -3 (c-CASP3) in nicotinamide-treated cells were examined via western blotting. (**e**) Densitometry analysis of BAX, BAD, cleaved caspase-9 (c-CASP9) and -3 (c-CASP3) was quantitated using ImageJ. The values were normalized to the levels of β-actin. The data represent the mean ± SD (**P < 0.01) Abbreviations: ER - Endoplasmic Reticulum, p - phosphorylation, WB - western blot.

This finding prompted us to analyze and organize a novel apoptosis-related interaction network induced by nicotinamide. Fifty mRNAs and proteins concordantly expressed were enriched based on the KEGG pathway database. Of these combinatory proteomic and transcriptomic outcomes and combined literature-based analyses (Fig. 7a and b), we reconstructed the nicotinamide induced p53 (TP53) related-apoptotic pathway, in which the acetylated form of p53 (TP53) sequentially activates not BAX, the well-established main factors involved in apoptosis, but PUMA (BBC3) and BAD unexpectedly^34^, which are subsequently re-located in the mitochondrial membrane where they bind to BCL2 and induce the extra-mitochondrial release of cytochrome *c* (CYCS) to the cytoplasm^35, 36^. Additional RT-PCR assays also showed elevated PUMA (BBC3) and BAD mRNA expression to validate the results from transcriptomic analysis (Fig. 7c). Released cytochrome *c* (CYCS) eventually results in the recruitment, processing and activation of pro-caspase-9 (pro-CASP9) into cleaved caspase-9 (c-CASP9) and subsequently induces the cleavage of caspase-3 (CASP3), an executor of apoptosis (Fig. 7d and e). To determine how caspase plays an important role in nicotinamide-induced apoptosis, Z-VAD-FMK, pan-caspase inhibitor was treated in different conditions described as follows in Supplementary Fig. S10. As a result, we have shown that activation of the caspase-related signaling pathway is crucial for nicotinamide-induced apoptosis through WST-1 assay, flow cytometry and western blotting (Supplementary Fig. S10). XIAP, which represses apoptosis solely through caspase inhibition, was also decreased in the present study and reflects the increased expression of SMAC/DIABLO increasingly released from the mitochondria after nicotinamide treatment, which neutralizes the caspase-inhibitory properties of the IAP family of proteins, particularly XIAP^37^.

In the present study, the mitochondrial fission protein Drp-1 (DNM1L) was also up-regulated at the proteomic levels. Drp-1 (DNM1L) is typically localized at the surface of mitochondria, and its overexpression increases the number of mitochondrial division events, which mediate apoptosis; thus, it is used to assess the role of mitochondrial division in apoptosis^38^.

In addition to apoptosis, the results of the interaction network analysis indicated the concomitant up-regulation of ER stress-related factors, including inositol-requiring protein-1 (IRE1, ERN1), binding immunoglobulin protein (Bip, HSPA5), activating transcription factor 6 (ATF6), glucose regulated protein 58 (GRP58, PDIA3), GRP94 (HSP90B1), GRP170 (HYOU1), growth arrest and DNA damage 153 (GADD153 and DDIT3), endoplasmic reticulum resident protein 44 (ERP44), ERp70 (PDIA4) and homocysteine-responsive endoplasmic reticulum-resident ubiquitin-like domain member 1 protein (HERPUD1), which partially overlapped with apoptosis (Fig. 3).

Previous studies have also suggested ER stress-induced apoptosis^39^. This finding prompted us to perform additional enrichment analyses to generate a combined interaction network between apoptosis and ER stress following nicotinamide treatment. ER-stress triggers the unfolded protein response (UPR), distinguished by the action of three signaling proteins, namely, IRE1 (ERN1), protein kinase RNA (PKR)-like ER kinase (PERK, EIF2AK3), and ATF6. Specifically, activated IRE1 (ERN1) promotes a phosphorylation cascade that ultimately activates Jun amino-terminal kinase (JNK, MAPK8)^40^. In this interaction network analysis combined with western blotting and RT-PCR, the IRE1 (ERN1) and JNK (MAPK8) axis was activated, which ultimately activates BAD^41^, a major component of apoptosis that promotes the leakage of cytochrome *c* (CYCS) into the cytoplasm (Supplementary Fig. S11). Thus, taken together, we concluded that nicotinamide induces apoptosis through the mitochondrial pathway and ER stress.

## Discussion

The current findings highlight how the combined-omics with transcriptome and high-throughput proteomics and systematic networking analysis may provide a better understanding of how nicotinamide modifies molecular signaling mechanisms and generates an integrated picture of the complex rewiring of the critical pathways away from tumor growth in TNBC cells. These findings provide evidence that nicotinamide primarily targets SIRT1 for negative regulation in cancer cell survival, which eventually triggers 1) S phase arrest through the restoration of RB and p53–p21 (TP53-CDKN1A) axis based activity and G2/M phase disturbances through the FOXM1-cyclin B1 (CCNB1) axis, 2) the execution of apoptosis and 3) the widespread dysfunction of DNA damage repair. Overall, systematic p53 (TP53) acetylation and SIRT1 inactivation initially play a pivotal role and eventually rewire downstream global interaction networks toward anti-tumor growth through the arrest of cell cycle progression, while promoting pro-cellular death conditions via the dysfunction of DNA damage repair and pro-apoptotic actions.

In the cell cycle progression pathway, the attenuation of the mitotic cell cycle checkpoint is regulated by the Aurora A (AURKA), PLK1 and cyclin B1(CCNB1)-mediated pathways accompanied by the down-regulation of multiple genes implicated in the mitotic spindle checkpoint, such as BUB1, MAD2L1, KIF2A, CDCA and SPC proteins. In addition, these expression changes are consistent with the changes of many other genes involved in chromosome segregation, centrosome function, cytokinesis, and the transition into and progression through mitosis.

The current findings also indicated the combined modification of broad apoptotic and ER stress-related genes and proteins. The observational data elucidates two major signaling mechanisms that trigger apoptosis: 1) ER stress inducible apoptosis via the IRE1 (ERN1) and JNK (MAPK8) pathway and 2) SIRT1 and the p53 (TP53) axis following nicotinamide treatment.

In addition to the activation of anti-tumor growth signaling pathways, we identified globally dysregulated functions of the DNA repair pathway induced by nicotinamide administration. These findings may ultimately lead to the application of nicotinamide to enhance chemotherapeutic benefits, such as the PARP1 inhibitor, olaparib, which disturbs repair functions for damaged DNA as a result of the nicotinamide-mediated disruption of major repair genes, including BRCA1, BRCA2, ATR, ATM and PARP1, in the multi-omic analysis in the present study.

Moreover, the expression of 21 of 150 proteins was discordant compared with their relevant mRNA expression (14.0%). We propose that this effect may reflect multiple post-translational modifications (PTM) that play crucial roles in the regulation of protein functions, including phosphorylation, ubiquitination or acetylation^42^. The follow-up integrated proteomic analysis of PTM will further the holistic view of the interaction networks in TNBC cells treated with nicotinamide.

In summary, these data provide global molecular evidence for the crucial role of nicotinamide in the induction of signaling alterations toward the anti-tumor growth and pro-death of tumor cells in TNBC by combining transcriptomic and high-throughput proteomic analysis. To our knowledge, we reported the first combinatory multi-omics and interaction networking study based on a text-based mining approach that aimed to understand the therapeutic potential of nicotinamide. These findings will provide more apparent knowledge for the future application of the agent in TNBC as the ONTRAC clinical trial indicated optimistic beneficial outcomes.

## Materials and Methods

### Cell line culture and proliferation assay

The TNBC cell line MDA-MB-231 was kindly provided by Prof. Moon Hyeong-Gon (Seoul National University Hospital) and cultured in Dulbecco’s modified Eagle’s medium (Hyclone) supplemented with 10% FBS (Hyclone) and 1% antibiotic-antimycotic solution (Gibco). The cells were seeded in triplicate onto 96-well culture plates. After 24 hours (h) of cultivation, the cells were treated with various concentrations of nicotinamide (Sigma-Aldrich) in a dose-dependent manner and Z-VAD-FMK, a pan-caspase inhibitor (Novus). After incubation for 0, 24 and 48 h, the cell viability was assessed using Premix WST-1 (Takara) measured at a wavelength of 450–650 nm.

### Colony formation assay

MDA-MB-231 cells were seeded onto 6-well plates at 1,000 cells per well. Following incubation for 11 days, the colonies were fixed with 4% formaldehyde (Junsei) in PBS for 20 min and stained with 0.1% crystal violet (Sigma-Aldrich) in PBS for 30 min. The number of stained colonies was counted to compare the colony formation ability (surviving colonies >50 cells per colony).

### Detection of apoptosis and cell cycle via flow cytometry

To evaluate nicotinamide-induced apoptosis, the MEBCYTO Apoptosis kit (MBL) was used. MDA-MB-231 cells were collected, and Annexin-FITC and propidium iodide (PI) were added to the cells. The cells were mixed with the reagents and incubated at room temperature in the dark for 5–15 min. Analysis using a FACSCalibur flow cytometer (Becton Dickinson) was performed to discriminate apoptotic cells.

To assess the effects of nicotinamide on the cell cycle, cells with or without nicotinamide treatment were fixed in cold ethanol for 24 h. Prior to detection, the ethanol was removed, and the cells were resuspended in staining buffer [50 μg/ml PI (Sigma- Aldrich) and 50 μg/ml RNase in PBS)] for 20 min at room temperature. The samples were analyzed with a FACSCalibur flow cytometer (Becton Dickinson) to determine the cell cycle distribution.

### Western blot analysis

The cells were lysed in a T-PER buffer (Thermo Scientific) that contained protease inhibitor cocktail (Roche), and 20–40 μg of the cell lysates were separated using SDS-PAGE and transferred to polyvinylidene difluoride (PVDF) membranes (Millipore). The blots were blocked in 5% skim milk and probed with antibodies against the appropriate primary antibodies (Table S1) for 18 h. After washing, the blots were incubated with horseradish peroxidase-conjugated secondary antibodies and visualized using ECL detection reagent (Elpis). The densitometric analysis of the protein expression was quantitated using ImageJ software (National Institutes of Health).

### Immunofluorescence

MDA-MB-231 cells were grown on coverslips at 70% confluence. Immunostaining was performed on cells fixed in 4% formaldehyde in PBS for 10 min. The primary antibody anti-p-γ-H2AX was used to assess DNA damage (Cell Signaling Technology). The secondary antibody included Alexa Fluor 488-conjugated goat anti-Rabbit IgG antibody (Invitrogen). Nuclear counterstaining with Hoechst 33342 (Thermo Scientific) was performed after the removal of excess secondary antibody. Immunostaining was visualized using a Confocal-A1 microscope (Nikon).

### RNA extraction and RT-PCR

Total RNA was isolated from MDA-MB-231 cells using TRIzol reagent (Invtrogen) according to the manufacturer’s instruction. An amount of 2 μg of total RNA was used in the reverse transcription reaction. The first strand cDNA was synthesized using standard random priming method with Moloney Murin Leukemia Virus reverse transcripts (Promega) and RNase inhibitor (Promega). After cDNA was synthesized, the primers of target genes were employed and HIPI Plus Master Mix (Elpis) was used to amplify the genes. The primer sequences for RT-PCR were listed in Supplementary Table S8.

### RNA isolation and cDNA synthesis

Total RNA was isolated from MDA-MB-231 cells using TRIzol reagent (Invitrogen). The total RNA quality and quantity were validated on a NanoCrop1000 spectrometer (Thermo Scientific) and Bioanalyzer 2100 (Agilent Technologies). We used the TruSeq RNA library preparation kit (Illumina). We constructed the Illumina-compatible libraries according to the manufacturer’s instructions. Additional information is provided in the supplementary experimental procedures.

### RNA library preparation and sequencing

To construct cDNA libraries using the TruSeq RNA library kit (Illumina), 1 µg of total RNA was used. The libraries were quantified using qPCR according to the qPCR Quantification Protocol Guide and qualified using an Agilent Technologies 2100 Bioanalyzer (Agilent Technologies). To estimate the expression levels, the aligned reads were counted for each gene using featureCounts v.1.4.6-p4^43^. Differentially expressed genes were estimated using DESeq v.1.25.0^44^, and a gene set enrichment analysis was performed using GSEA2 v.2.2.2^45^. The detailed process of the data analysis is described in the supplementary experimental procedures.

### Proteome sample preparation

MDA-MB-231 cells were lysed in SDS-lysis buffer. After alkylation and reduction, the proteins were digested using an FASP procedure as previously described^46^. Tandem mass tag (TMT) 6-plex labeling was performed according to the manufacturer’s instructions, with modifications. After the labeled peptides were pooled, the samples were separated using high-pH reversed phased liquid chromatography. Additional information is provided in the supplemental experimental procedures.

### Mass spectrometry analysis and data processing

The fractionated peptides were analyzed using Quadrupole Orbitrap mass spectrometry, consisting of a Q-exactive Plus (Thermo Scientific) coupled to an Ultimate 3000 RSLC system (Dionex) via a nano electrospray source as previously described, with modifications^46, 47^. Protein identification and quantification were performed using Proteome Discoverer 2.1 software interfaced with the SEQUEST-HT search engine based on the Human UniProt database (December 2014; 88,657 entries). The detailed process of data analysis is described in the supplementary experimental procedures.

### Data analysis

MS raw files were processed using Proteome Discoverer 2.1 software interfaced with the SEQUEST-HT search engine based on the Human UniProt database (December 2014, 88,657 entries), which included forward and reverse protein sequences and common contaminants. Searches were performed using a 10-ppm precursor ion tolerance for total protein level analysis. The MS/MS ion tolerance was set to 20 ppm. TMT tags on lysine residues and peptide N termini (+229.163 Da) and carbamido-methylation of cysteine residues (+57.021 Da) were set as fixed modifications, whereas the oxidation of methionine residues (+15.995 Da) was set as a variable modification. The false discovery rate (FDR) for all PSM, peptides, and protein assignments was determined using the Percolator software package. Reporter ion quantification was performed in the MS2 channel with a 20-ppm mass tolerance. Only PSMs that contained all 6 reporter ions were considered. Each reporter ion channel was summed across all quantified proteins and normalized with the assumption of equal protein loading for all 6 samples.

### Bioinformatics and statistical analysis

The statistical analyses were performed using Student’s *t*-tests. The threshold of significance was set at *P* < 0.05. A bioinformatics analysis was performed using Perseus software^48^. Statistical analysis of the proteome was performed based on the logarithmized intensities of the TMT-reporter ions. To identify significantly expressed proteins, we performed a two-sample *t*-test analysis using a Benjamini-Hochberg FDR cutoff of 0.05. The gene ontology of the analyzed proteins was explicated using the DAVID bioinformatics tool (http://david.abcc.ncifcrif.gov/). The pathways were analyzed using the KEGG database. Interaction network models were constructed using Cytoscape ver3.4.0^49^ and Illustrator (Adobe). All proteomic datasets were submitted to the ProteomeXchange Consortium (http://proteomecentral.proteomechange.org) via the PRIDE partner repository^50^ (Accession number PXD005304). All transcriptomic datasets were submitted to the SRA site (https://www.ncbi.nlm.nih.gov/sra) and received accession number (SRP093217).

## Electronic supplementary material

Dataset1 & Dataset2
