# Supplementary material for: Reconstruction of pathway modification induced by nicotinamide using multi-omic network analyses in triple negative breast cancer
Source: Sci Rep. 2017 Jun 14;7:3466. doi: 10.1038/s41598-017-03322-7 (PMC5471278; doi:10.1038/s41598-017-03322-7)
Supplement: Supplementary file 1 — Dataset1 & Dataset2 [file 41598_2017_3322_MOESM1_ESM.zip › Supplementary dataset_NA/Supplementary_Information_Final_revised_ver.pdf]

# Reconstruction of pathway modification induced by nicotinamide using multi-omic network analyses in triple negative breast cancer

Ji Young Kim[1*], Hyebin Lee[2*], Jongmin Woo[3], Wang Yue[1], Kwangsoo Kim[4], Seongmin Choi[4], Ja-June Jang[1], Youngsoo Kim[3], In Ae Park[1], Dohyun Han[5**], Han Suk Ryu[1**]

[1]Department of Pathology, Seoul National University Hospital, Seoul National University College of Medicine, Seoul, South Korea,
[2]Department of Radiation Oncology, Kangbuk Samsung Hospital, Sungkyunkwan University School of Medicine, Seoul, Korea,
[3]Department of Biomedical Sciences, Seoul National University College of Medicine, Seoul, South Korea,
[4]Division of Clinical Bioinformatics, Biomedical Research Institute, Seoul National University Hospital, Seoul, Korea,
[5]Proteomics Core Facility, Biomedical Research Institute, Seoul National University Hospital, Seoul, Korea.

Correspondence to:
Han Suk Ryu. M.D., Ph.D.
Department of Pathology, Seoul National University Hospital
Seoul National University College of Medicine, 101 Daehak-ro, Jongno-gu
Seoul 110-744 (Korea)
E-Mail: karlnash@naver.com

Dohyun Han. Ph.D.
Proteomics Core Facility, Biomedical Research Institute, Seoul National University Hospital
Seoul National University College of Medicine, 101 Daehak-ro, Jongno-gu
Seoul 110-744 (Korea)
E-Mail: hdh03@snu.ac.kr

[*] Ji Young Kim and Hyebin Lee contributed equally to this work.
[**] Han Suk Ryu and Dohyun Han jointly supervised this work.

**Supplementary Figure Legends**

Supplementary Figure S1. **Nicotinamide inhibits cell growth in TNBC cells.** (a) MDA-MB-468 and BT20 cells were treated with 15, 25 and 45 mM nicotinamide. Dose-dependent cytotoxic effects were assessed every 24 and 48 h using a WST-1 assay. The data represent the mean ± SD (**$P$<0.01). The cell morphology of MDA-MB-468 and BT20 cells treated with 25 mM nicotinamide for 48 h via (b) light microscopy (original magnification x 100) and (c) H&E staining (original magnification x 100).

Supplementary Figure S2. **Nicotinamide induces cell cycle arrest and apoptosis in TNBC cells.** Representative flow cytometry for the effect of 25 mM nicotinamide on MDA-MB-231, MDA-MB-468 and BT20 cells for 48 h. (a) Analysis of subpopulation of MDA-MB-231, MDA-MB-468 and BT20 cells in cell cycle phases. (b) Bar graph expressed as percent cells in different phases of cell cycle. The data represent the mean ± SD (**$P$<0.01). (c) Cells were subsequently labeled with Annexin V and PI and analyzed via flow cytometry. (d) Bar graph represents apoptotic cell population of MDA-MB-231, MDA-MB-468 and BT20 cells. The data represents the mean ± SD (*$P$<0.05, **$P$<0.01).

Supplementary Figure S3. **Nicotinamide affects acetylation of p53 through SIRT1.** (A) MDA-MB-231 cells were treated with nicotinamide 0, 15 and 25 mM for 48 h. SIRT1, acetylated p53, total p53 (TP53), and β-actin were measured via immunoblotting in whole cell extracts. β-actin was used as a loading control. (B) Densitometry analysis of SIRT and ac-p53 was quantitated using ImageJ. Values were normalized to the levels of β-actin and total p53. The data represent the mean ± SD (**$P$<0.01).

Supplementary Figure S4. **Correlation of quantified transcriptome and proteome in nicotinamide treated and untreated MDA-MB-231 cells.** (a) Comparison between the biological replicates of transcriptome experiments of nicotinamide treated (Treatment 1-3) and untreated (Control 1-3) cells. Each dot indicates one gene. The Pearson correlation coefficients ($R^2$) are represented in each scatterplot. (b) Comparison between biological replicates of TMT-based quantitative proteomic experiments of nicotinamide treated (Treatment 1-3) and untreated (Control 1-3) cells. Each dot indicates one protein group. The Pearson correlation coefficients ($R^2$) are represented in each scatter plot.

Supplementary Figure S5. **Accuracy of TMT quantification.** Reporter ion intensities of each channel were calculated using Proteome Discoverer 2.1. Coefficient of variation (CV) values of the biological replicates are indicated as box plots. The red lines indicate 20% of the CV value.

Supplementary Figure S6. **Gene ontology and pathway enrichment analysis for quantified transcriptome and proteome in nicotinamide treated cells.** Gene ontology (upper panel) and KEGG pathway (lower panel) enrichment analyses were independently performed in up-regulated genes and proteins (left) and down-regulated genes and proteins (right) in response to nicotinamide. Logarithmic corrected $P$-values for significant overrepresentation are indicated.

Supplementary Figure S7. **Analysis of the expression of cell cycle related genes in nicotinamide treated cells.** (a) The expression of cell cycle related proteins cyclin A (CCNA), CDK2, cyclin B1 (CCNB1), CDC2 and p21 (CDKN1A) was examined via western blotting. (b) Densitometry analysis of cyclin A (CCNA), CDK2, cyclin B1 (CCNB1), CDC2 and p21 (CDKN1A) was quantitated using ImageJ. Values were normalized to the levels of β-actin. Densitometric analysis was performed using ImageJ. The data represent the mean ± SD (**$P$<0.01). (c) The mRNA expression of cyclin A (CCNA), cyclin B1 (CCNB1), SIRT1, p21 (CDKN1A) and FOXO3 was detected by RT-PCR. PCR control indicates negative control for RT-PCR mix. Expression of GAPDH was measured as a control for RNA integrity.

Supplementary Figure S8. **Nicotinamide suppresses DNA replication-related genes.** The mRNA expression of MCM2, 3, 6 and CDC6 was detected by RT-PCR. PCR control indicates negative control for RT-PCR mix. Expression of GAPDH was measured as a control for RNA integrity.

Supplementary Figure S9. **Nicotinamide decreased genes expression involving DNA damage response.** The mRNA expression of ATM, ATR, BRCA1, RAD51, FANCD2 and BRCA2 was detected by RT-PCR. PCR control indicates negative control for RT-PCR mix. Expression of GAPDH was measured as a control for RNA integrity.

Supplementary Figure S10. **Inhibition of nicotinamide-induced apoptosis by the pan-caspase inhibitor treatment in MDA-MB-231 cells.** Cells were pretreated with Z-VAD-FMK (20 μM) for 1h, and treated further with nicotinamide (25 mM). (a) Cell proliferation was measured by the WST-1 assay. The data represents the mean ± SD (**$P$<0.01). (b) Cells were subsequently labeled with Annexin V and PI and analyzed via flow cytometry. MDA-MB-231 cells pretreated with Z-VAD-FMK (20 μM) and treated with nicotinamide 25 mM for 48 h. (c) The expression of cleaved caspase-3 (c-CASP3) and PARP1 was examined via western blotting. β-actin was used as a loading control.

Supplementary Figure S11. **Nicotinamide induces ER stress and promotes activation of unfolded protein response (UPR).** (a) MDA-MB-231 cells were treated with nicotinamide for 48 h. Whole lysates were subjected to immunoblot analysis to detect ER stress-related proteins, ATF6, IRE1 (ERN1), JNK (MAPK8), p-JNK, PERK (EIF2AK3) and p-PERK. (b) Densitometry analysis of ATF6, IRE1 (ERN1), JNK (MAPK8), p-JNK, PERK

(EIF2AK3) and p-PERK was quantitated using ImageJ. The values were normalized to the levels of β-actin, JNK and PERK. The data represent the mean ± SD (*$P<0.05$, **$P<0.01$). (c) The mRNA expression of IRE1, ATF6 and CHOP was detected by RT-PCR. PCR control indicates negative control for RT-PCR mix. Expression of GAPDH was measured as a control for RNA integrity.

Supplementary Figure S12. Raw data of Western blots. (a) Unprocessed scan of Western blot for ac-p53, p53, SIRT1, RB, p-RB, ac-RB and β-actin. (b) Unprocessed scan of Western blot for p21 (CDKN1A), cyclin A (CCNA), cyclin B1 (CCNB1), CDK2, CDC2 and β-actin. (c) Unprocessed scan of Western blot for γ-H2AX (H2AFX) and β-actin. (d) Unprocessed scan of Western blot for PARP1, ATR, ATM and β-actin. (e) Unprocessed scan of Western blot for BAX, BAD, cleaved caspase 9 (c-CASP9), cleaved caspase 3 (c-CASP3) and β-actin. (f) Unprocessed scan of Western blot for PARP1, cleaved caspase 3 (c-CASP3) and β-actin. (b) Unprocessed scan of Western blot for ATF6, IRE1 (ERN1), JNK (MAPK8), p-JNK, PERK (EIF2AK3) and p-PERK and β-actin. Abbreviations: ac - acetylation, p - phosphorylation

Supplementary Figure S13. Agarose gel electrophoresis of different DNA products. (a) Representative raw data of cyclin A (CCNA), MCM and RAD51. (b) Representative raw data of BRCA2, MCM3 and FANCD2. (c) Representative raw data of ATM and ATR. (d) Representative raw data of CDC6 and ATF6. (e) Representative raw data of IRE1 (ERN1) and MCM6. (f) Representative raw data of p21 (CDKN1A) and FOXO3. (g) Representative raw data of PUMA (BBC3), BAD and CHOP (GADD153). (h) Representative raw data of BRCA1, cyclin B (CCNB), SIRT1 and GAPDH. DNA ladder (Elpis). We used DNA size marker (1kb DNA ladder, Elpis). Abbreviation: c - PCR control

**Supplementary Table captions**

Supplementary Table S1. List of total quantified genes in RNA-Seq. This list includes the official gene symbol and description of the genes. In each sample, the FPKM values for each biological replicate are presented.

Supplementary Table S2. List of total identified protein groups. This list includes the UniProt accession number, official gene symbol, and protein name of the identified protein groups. The search results from Proteome Discoverer 2.1 based on SequestHT are also described.

Supplementary Table S3. List of differentially expressed genes. All significantly expressed genes with an adjusted *P*-value <0.05 and 2-fold changes are listed.

Supplementary Table S4. List of differentially expressed proteins in TMT-6 plex quantification. All differentially expressed proteins with a *P*-value <0.05 are listed. In each sample, the log2-transformed reporter ion intensity from each biological replicate is presented.

Supplementary Table S5. Geno ontology and pathway enrichment of proteome. Gene ontology annotation and KEGG pathway enrichment of proteome were performed using the DAVID bioinformatics resource (http://david.abcc.ncifcrf.gov) and KEGG pathway database, respectively. The *P*-value cut-offs for the gene ontology annotation and KEGG pathway enrichment were set to <0.05. The genes involved in each gene ontology and KEGG term are provided as official gene symbols.

Supplementary Table S6. Gene ontology and pathway enrichment of transcriptome. Gene ontology annotation and KEGG pathway enrichment were performed using the DAVID bioinformatics resource (http://david.abcc.ncifcrf.gov) and KEGG pathway database, respectively. The *P*-value cut-offs for the gene ontology annotation and KEGG pathway enrichment were set to <0.05. The genes involved in each gene ontology and KEGG term are provided as official gene symbols.

Supplementary Table S7. List of antibodies used for the western blot assay.

Supplementary Table S8. List of primers for RT-PCR.

## Materials and Methods

### RNA isolation and cDNA synthesis

MDA-MB-231 cells treated and non-treated with nicotinamide were extracted using TRIzol reagent (Invitrogen) according to the manufacturer's instructions. The total RNA quality and quantity were validated using a NanoDrop1000 spectrometer (Thermo Scientific) and Bioanalyzer 2100 (Agilent technologies). We constructed the Illumina-compatible libraries through the TruSeq RNA library preparation kit (Illumina) following the manufacturer's instructions. Briefly, mRNA was first purified using polyA selection. PolyA enriched total RNA from MDA-MB-231 cells was chemically fragmented and converted into single-stranded cDNA using random hexamer priming. Reverse transcription was subsequently performed to produce second-strand cDNA fragments available for the TruSeq library construction. After synthesis, short double-stranded cDNA fragments were connected with sequencing adapters, and appropriate fragments were separated via agarose gel electrophoresis. Finally, TruSeq RNA libraries were built via PCR amplification, quantified using qPCR according to the qPCR Quantification Protocol Guide and qualified using an Agilent Technologies 2100 Bioanalyzer (Agilent Technologies).

### RNA library preparation and sequencing

To construct cDNA libraries with the TruSeq RNA library kit (Illumina), 1 µg of total RNA was used. The protocol involved polyA-selected RNA extraction, RNA fragmentation, random hexamer primed reverse transcription and 100 nt paired-end sequencing using an Illumina HiSeq™ 2500 platform (Illumina). The libraries were quantified using qPCR according to the qPCR Quantification Protocol Guide and qualified using an Agilent Technologies 2100 Bioanalyzer (Agilent Technologies).

To increase the mapping quality, the raw RNA-Seq reads were trimmed according to their base quality scores, using Trimmomatic v.0.35 [1]. The trimmed reads were subsequently happed to the human reference genome hg19, using STAR v.2.4.1d [2]. To estimate the expression levels, the aligned reads were counted for each gene using FeatureCounts v.1.4.6-p4 [3]. The transcript counts in the gene level were calculated, and the relative transcript abundances were measured in FPKM (Fragments Per Kilobase of exon per Million fragments mapped). Differentially expressed genes were estimated using DESeq v.1.25.0 [4], following the removal of genes with a median read count <10 for the treatment and control groups, respectively. A gene set enrichment analysis was subsequently performed using GSEA2 v.2.2.2 [5] to examine the genes significantly enriched in manually curated pathways (c2.cp.v5.0[5,6]), motif gene sets (c3.all.v5.0), or gene ontology gene sets (c5.all.v5.0).

### Cell lysis and protein digestion

MDA-MB-231 cells were lysed in SDS-lysis buffer (4% SDS and 1 mM TCEP in 0.1 M Tris, pH 7.5) via 5 min sonication. After measurement of the protein concentration using a BCA reducing agent-compatible kit, 100 µg of proteins were precipitated overnight at -20°C using acetone. Proteins were digested via the FASP procedure as described with modifications [7,8]. Protein pellets were resolved in SDT buffer (4% SDS and 0.1 M DTT in 0.1 M TEAB pH 8.0) and loaded onto a 30 K Amicon filter (Millipore). The buffer was exchanged with UA solution (8 M urea in 0.1 M TEAB pH 8.5) via centrifugation at 14,000 x *g*. Reduced cysteines were alkylated with 50 mM IAA solution for 30 min at room temperature (RT) in the dark. Following the exchange of buffer with 50 mM TEAB, protein digestion was performed at 37°C overnight using a trypsin/LysC mixture at a 100:1 protein-to-protease ratio.

### Tandem mass tag labeling

Tandem mass tag (TMT) 6-plex labeling was performed according to the manufacturer's instructions, with modifications. Briefly, the peptide concentration was measured via a tryptophan assay [9]. TMT reagents (0.8 mg) were dissolved in anhydrous acetonitrile (ACN) of which 10 µl were added to the peptides (50 µg) in addition to acetonitrile to achieve a final acetonitrile concentration of approximately 30% (v/v). The non-treatment samples (control) were labeled with 126, 128 and 130, whereas the nicotinamide treated samples were labeled with 127, 129 and 131. For normalization and the labeling quality check, the peptides (500 ng) derived from ovalbumin were spiking into all channels. Following incubation at room temperature for 1 h, the reaction was quenched with hydroxylamine to a final concentration of 0.3% (v/v). The TMT-labeled samples were pooled at a 1:1:1:1:1:1 ratio. The sample was dried using a Speed-Vac and subjected to C18 solid-phase extraction (SPE) for desalting.

### Offline High-pH reverse-phased fractionation

The TMT-labeled peptide pooled mixtures were fractionated using Agilent 1290 bioinert HPLC (Agilent, Santa Clara, CA) equipped with an analytical column (4.6 x 250 mm, 5 µm). High-pH reverse-phase liquid chromatography was performed at a flow rate of 0.8 ml/min on a 60-min gradient using Solvent A (15 mM ammonium hydroxide in water) and solvent B (15 mM ammonium hydroxide in 90% ACN). The peptides were separated with a gradient of 5% to 35% acetonitrile at 0.2 ml/min. Ninety-six fractions were collected every minute

from 1 to 40 min and were non-contiguously concatenated into 12 fractions.

**LC-MS/MS analysis**

MS analysis was performed using Quadrupole Orbitrap mass spectrometers, Q-exactive plus (Thermo Scientific) coupled to an Ultimate 300 RSLC system (Dionex) via a nano electrospray source, as previously described with modifications [7,10]. The fractionated samples were separated on a 2-column system with a trap column and an analytical column (75 µm inner diameter, 50 cm length) with 240-min gradients from 7% to 32% acetonitrile at 300 nl/min and analyzed on the mass spectrometer. The column temperature was constantly set to 60°C using a column heater. The survey scans (350 to 1650 *m*/*z*) were acquired with a resolution of 70,000 at *m*/*z* 200. A top 20 method was used to select up to the 20 most abundant precursor ions with an isolation window of 1.2 *m*/*z*. The selected precursor ions were subjected to high-energy collisional dissociation (HCD) fragmentation at a normalized collision energy of 32 with a resolution of 35,000 at *m*/*z* 200. The maximum ion injection times for the full scan and MS/MS scan were 20 and 100 ms, respectively.

**Supplementary References**


1 Bolger, A. M., Lohse, M. & Usadel, B. Trimmomatic: a flexible trimmer for Illumina sequence data. *Bioinformatics* **30**, 2114-2120, doi:10.1093/bioinformatics/btu170 (2014).

2 Dobin, A. *et al.* STAR: ultrafast universal RNA-seq aligner. *Bioinformatics* **29**, 15-21, doi:10.1093/bioinformatics/bts635 (2013).

3 Liao, Y., Smyth, G. K. & Shi, W. featureCounts: an efficient general purpose program for assigning sequence reads to genomic features. *Bioinformatics* **30**, 923-930, doi:10.1093/bioinformatics/btt656 (2014).

4 Anders, S. & Huber, W. Differential expression analysis for sequence count data. *Genome Biol* **11**, R106, doi:10.1186/gb-2010-11-10-r106 (2010).

5 Subramanian, A. *et al.* Gene set enrichment analysis: a knowledge-based approach for interpreting genome-wide expression profiles. *Proc Natl Acad Sci U S A* **102**, 15545-15550, doi:10.1073/pnas.0506580102 (2005).

6 Xie, X. *et al.* Systematic discovery of regulatory motifs in human promoters and 3' UTRs by comparison of several mammals. *Nature* **434**, 338-345, doi:10.1038/nature03441 (2005).

7 Han, D., Jin, J., Woo, J., Min, H. & Kim, Y. Proteomic analysis of mouse astrocytes and their secretome by a combination of FASP and StageTip-based, high pH, reversed-phase fractionation. *Proteomics* **14**, 1604-1609, doi:10.1002/pmic.201300495 (2014).

8 Wisniewski, J. R., Zougman, A., Nagaraj, N. & Mann, M. Universal sample preparation method for proteome analysis. *Nat Methods* **6**, 359-362, doi:10.1038/nmeth.1322 (2009).

9 Wisniewski, J. R. & Gaugaz, F. Z. Fast and sensitive total protein and Peptide assays for proteomic analysis. *Anal Chem* **87**, 4110-4116, doi:10.1021/ac504689z (2015).

10 Han, D. *et al.* In-depth proteomic analysis of mouse microglia using a combination of FASP and StageTip-based, high pH, reversed-phase fractionation. *Proteomics* **13**, 2984-2988, doi:10.1002/pmic.201300091 (2013).

**Supplementary Figure. S1**

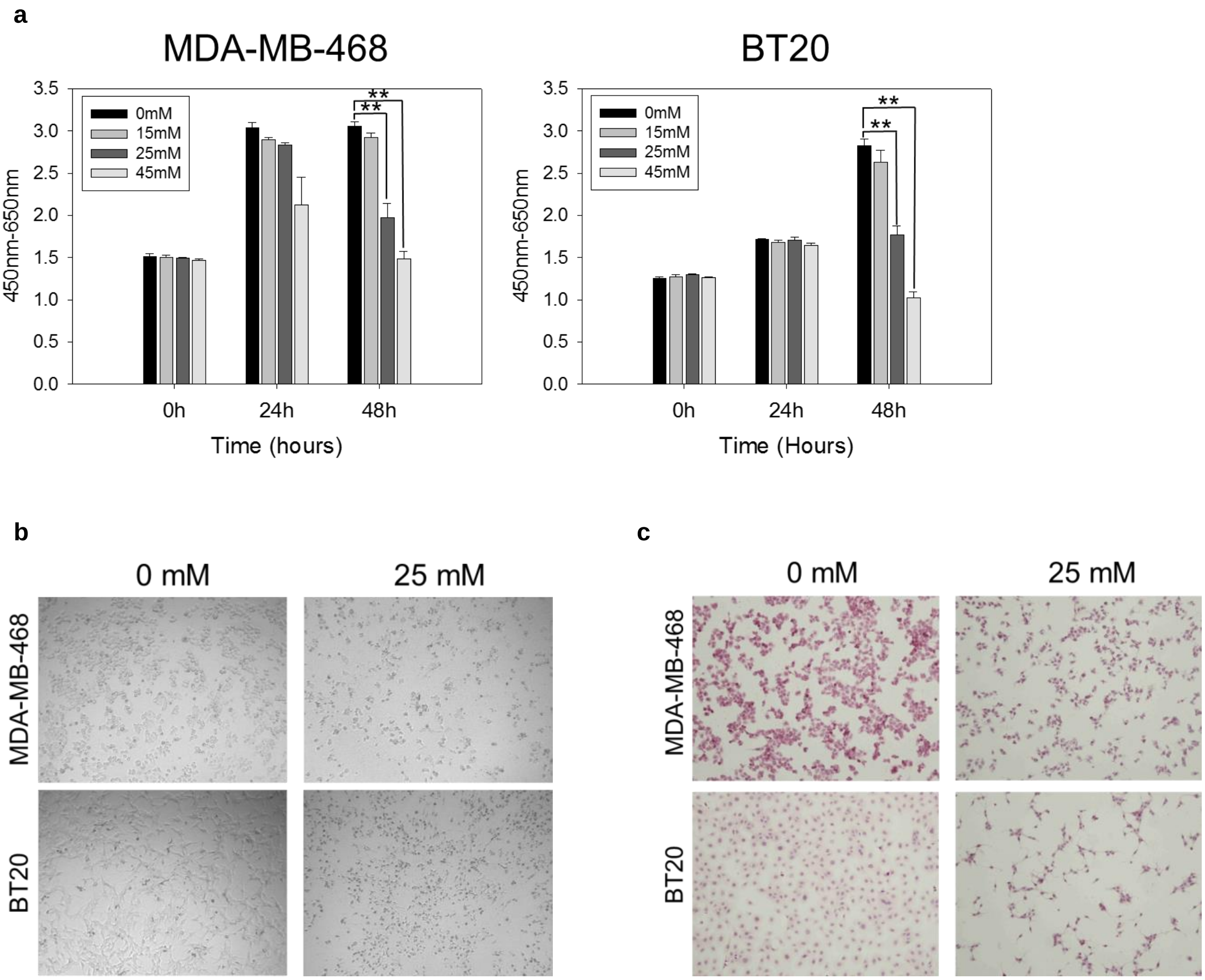

**Supplementary Figure. S2**

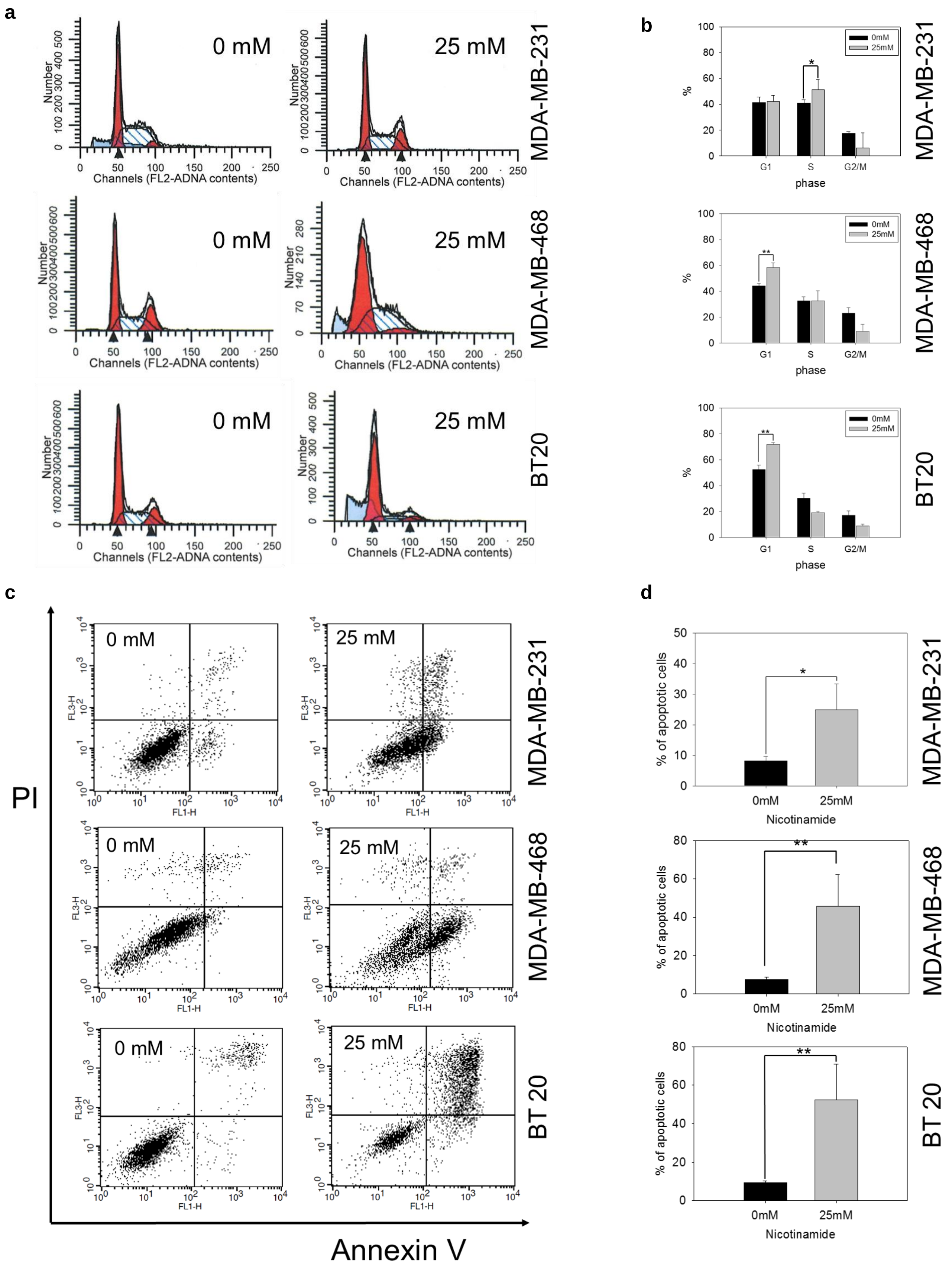

**Supplementary Figure. S3**

**a**

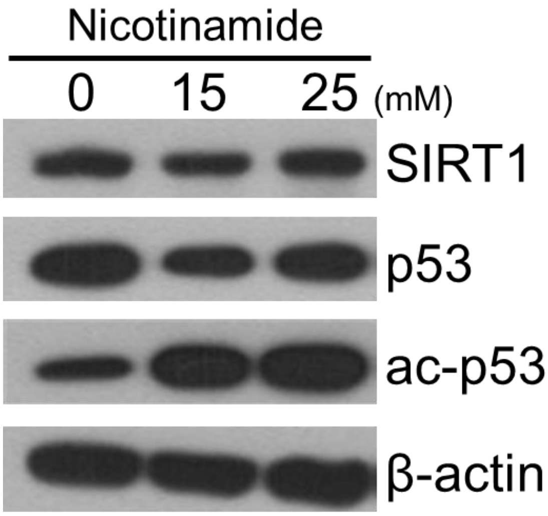


**b**

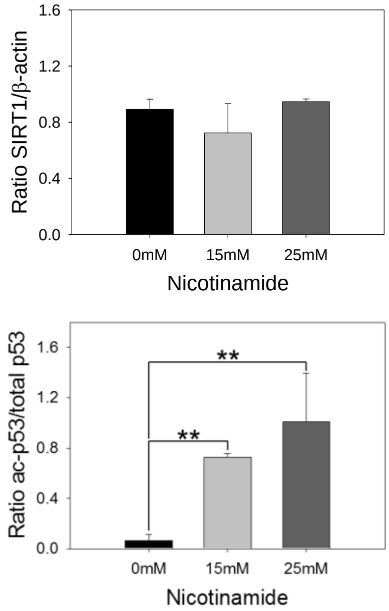

**Supplementary Figure. S4**

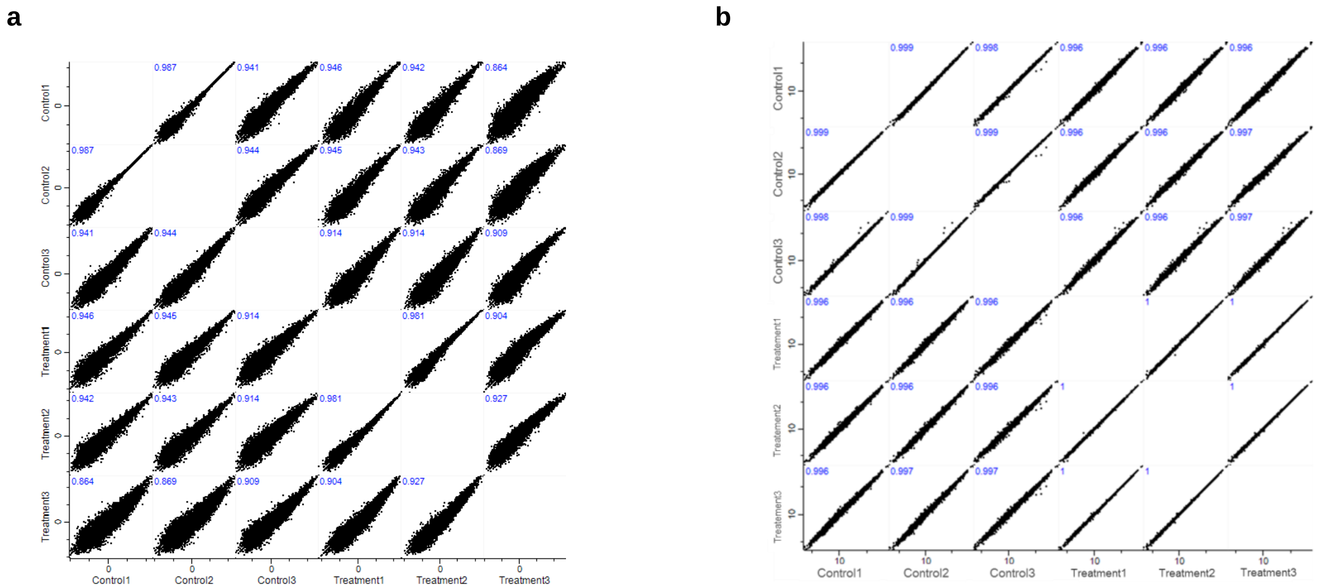

**Supplementary Figure. S5**

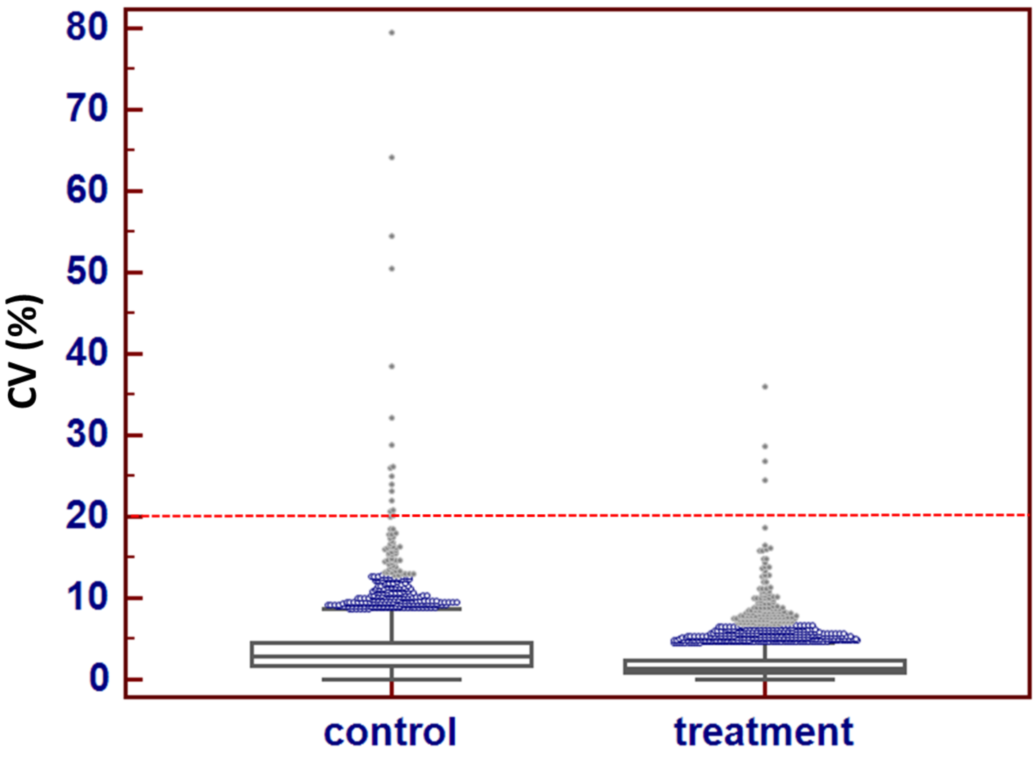

## Supplementary Figure. S6

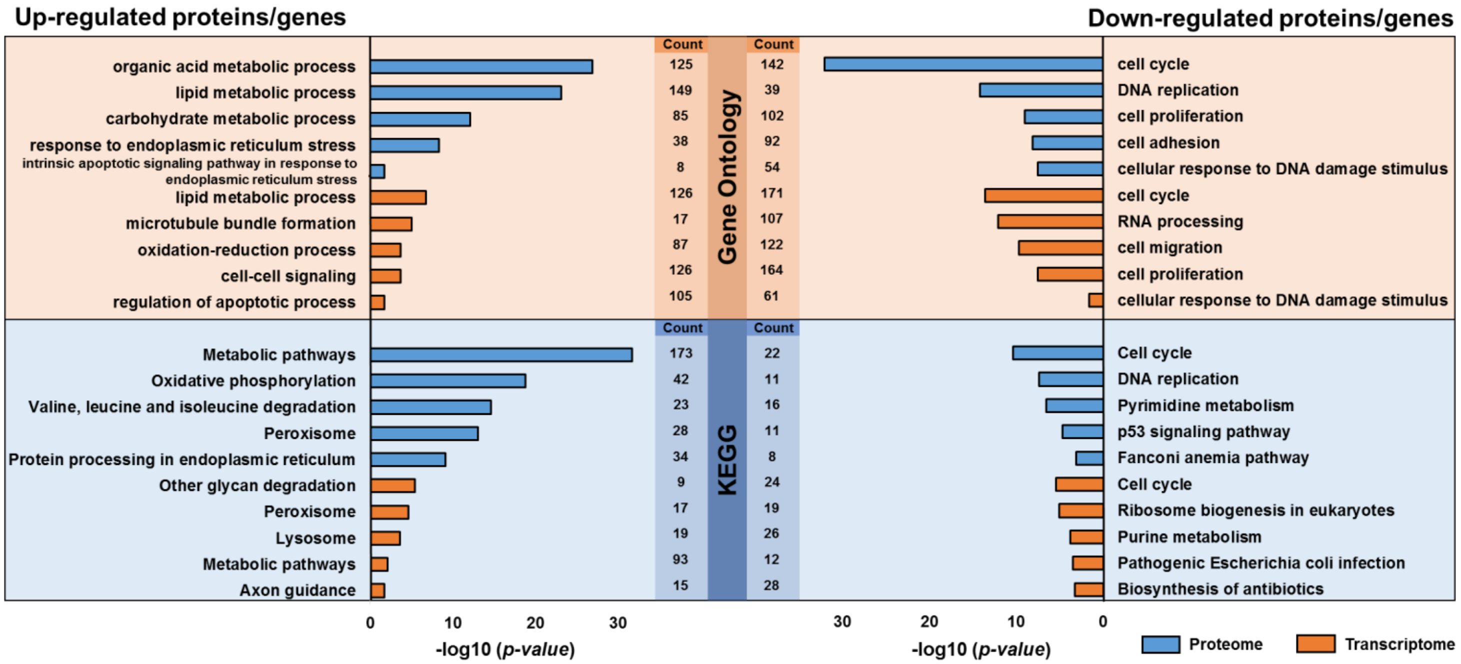

## Supplementary Figure. S7

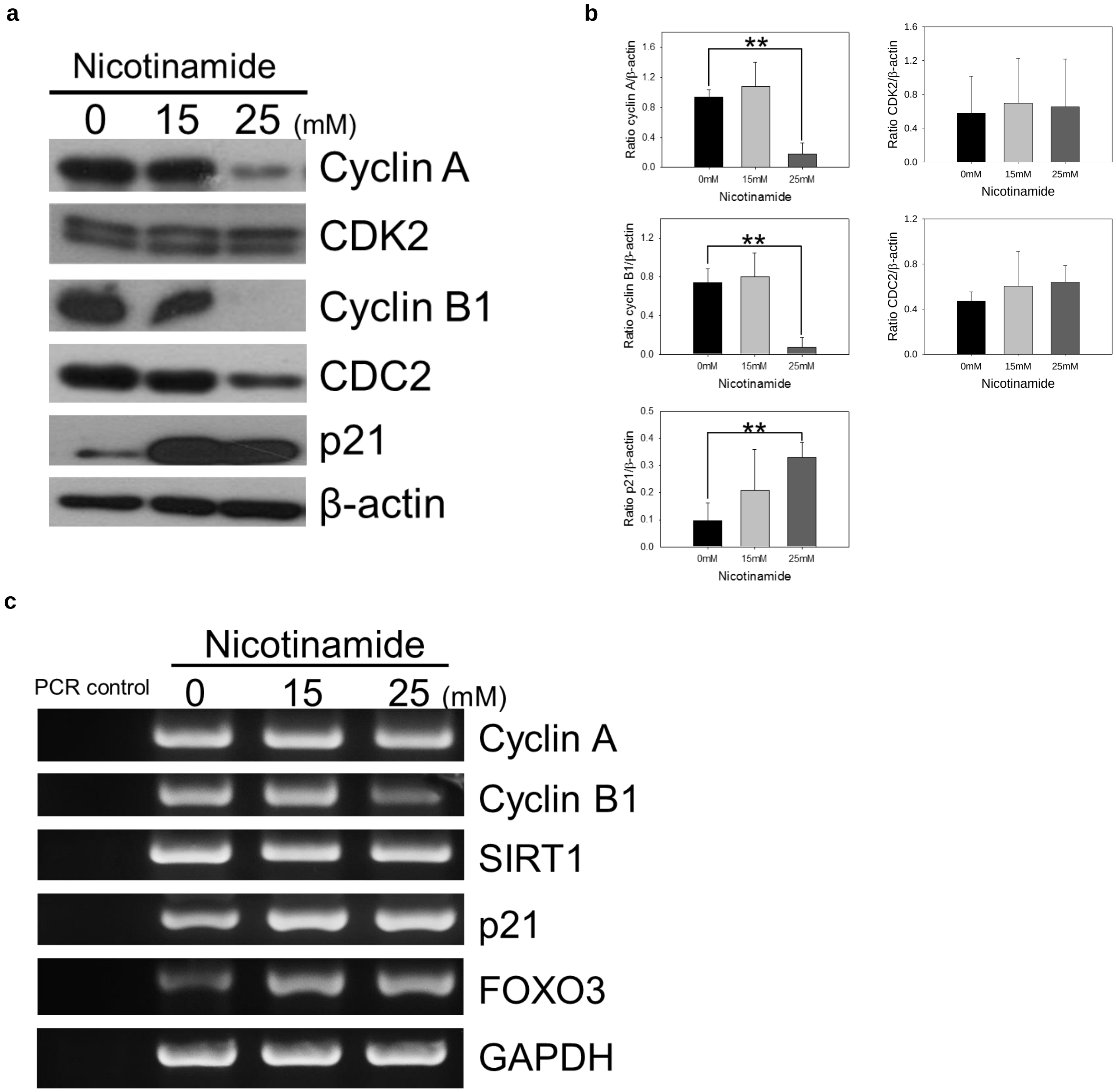

**Supplementary Figure. S8**

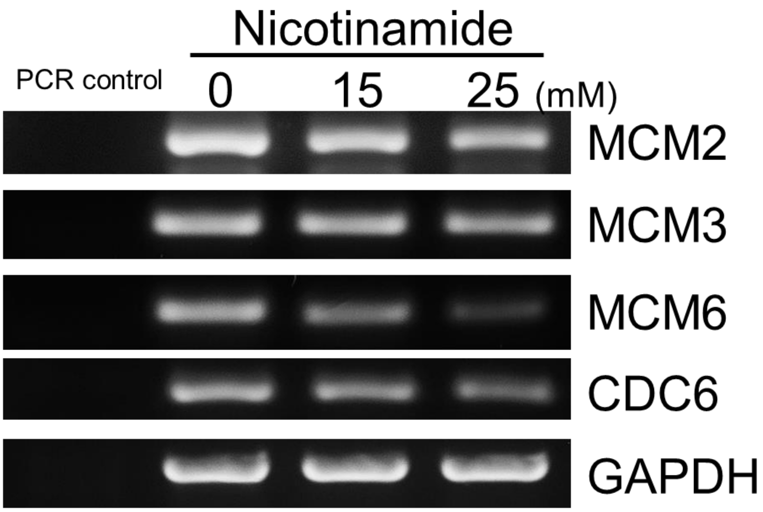

**Supplementary Figure. S9**

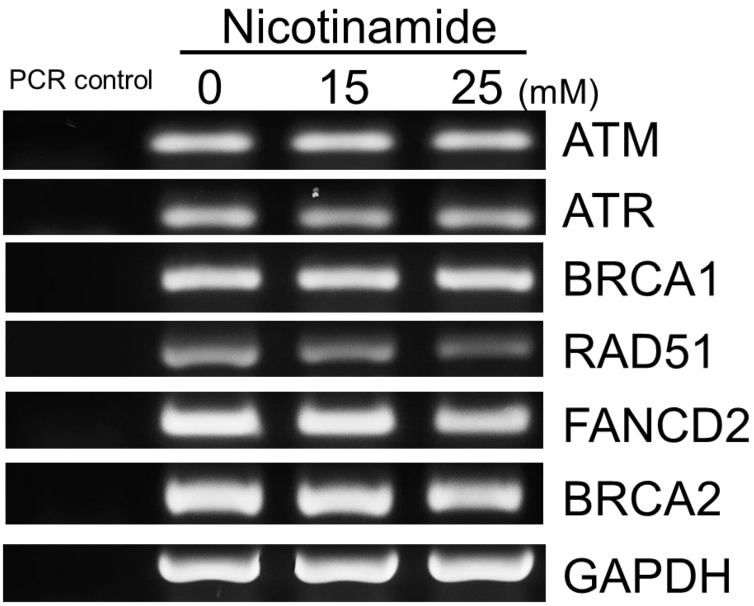

**Supplementary Figure. S10**

a

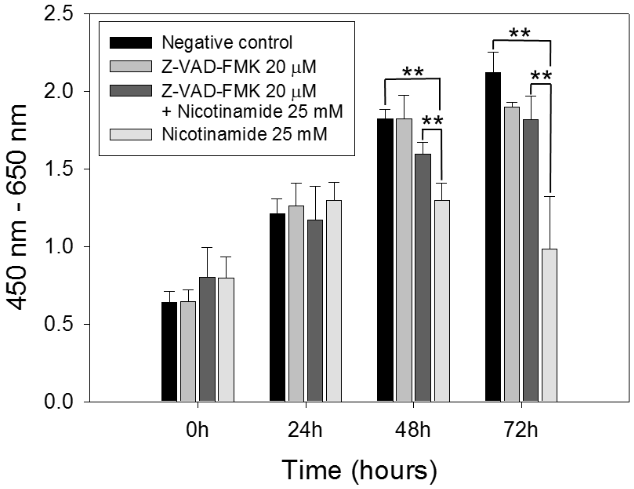


b

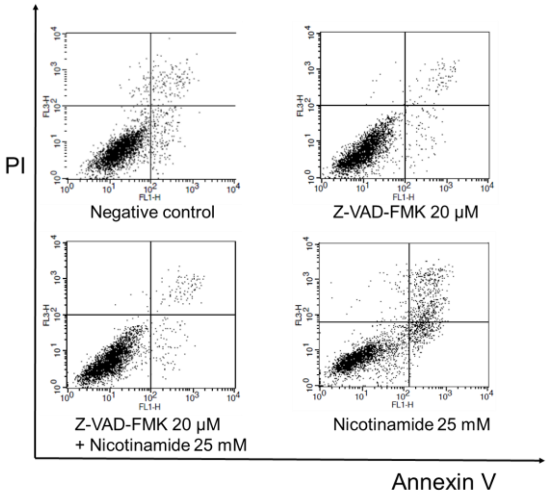


c

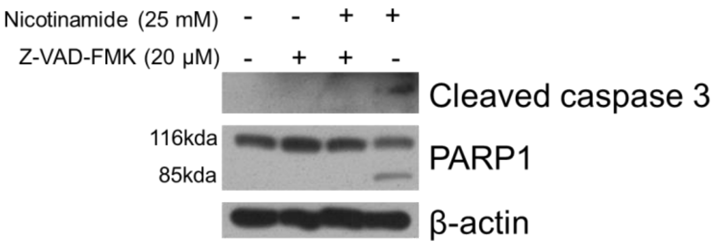

**Supplementary Figure. S11**

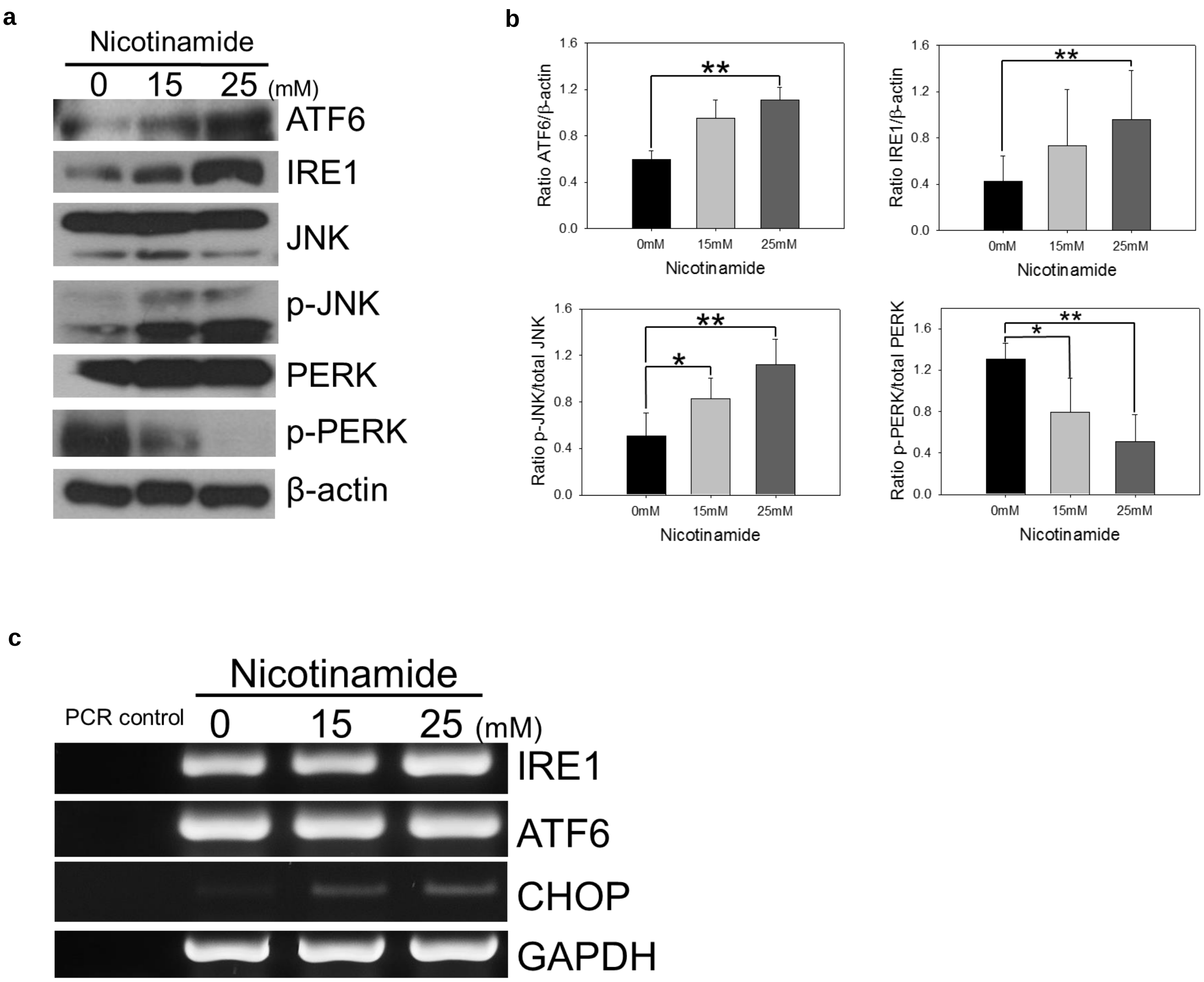

## Supplementary Figure. S12

a

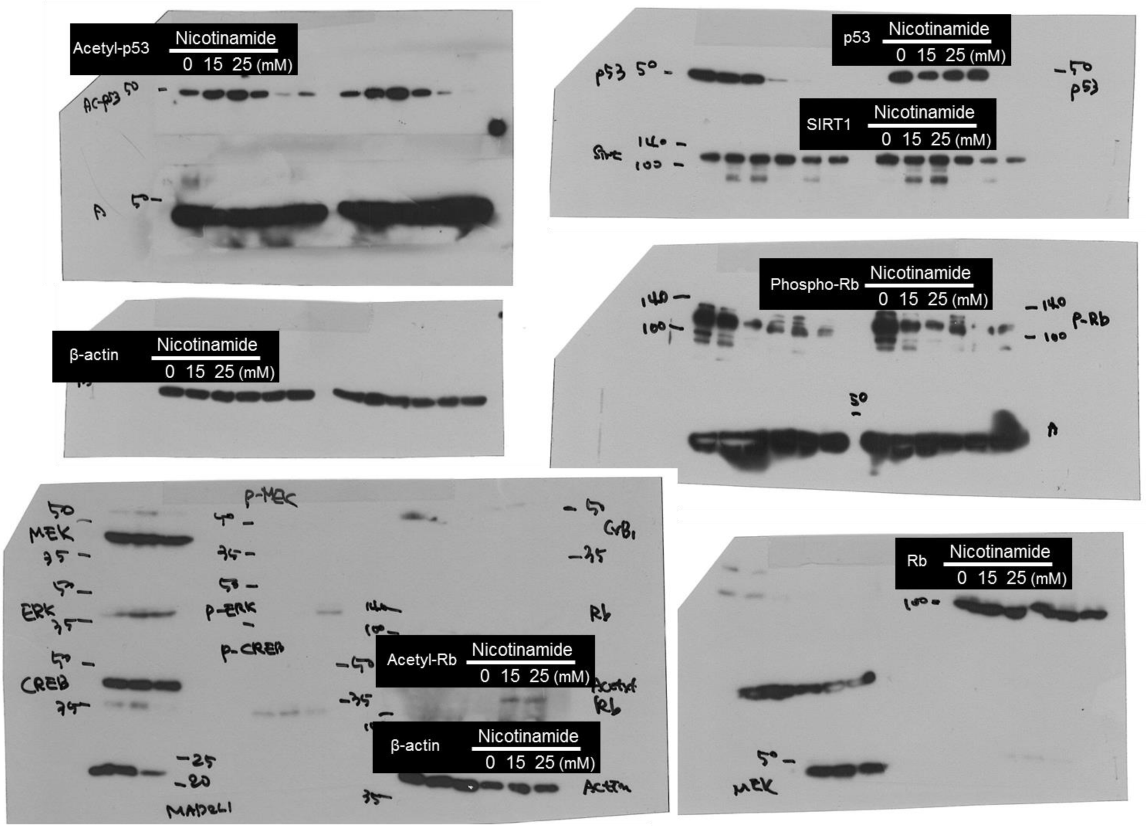


b

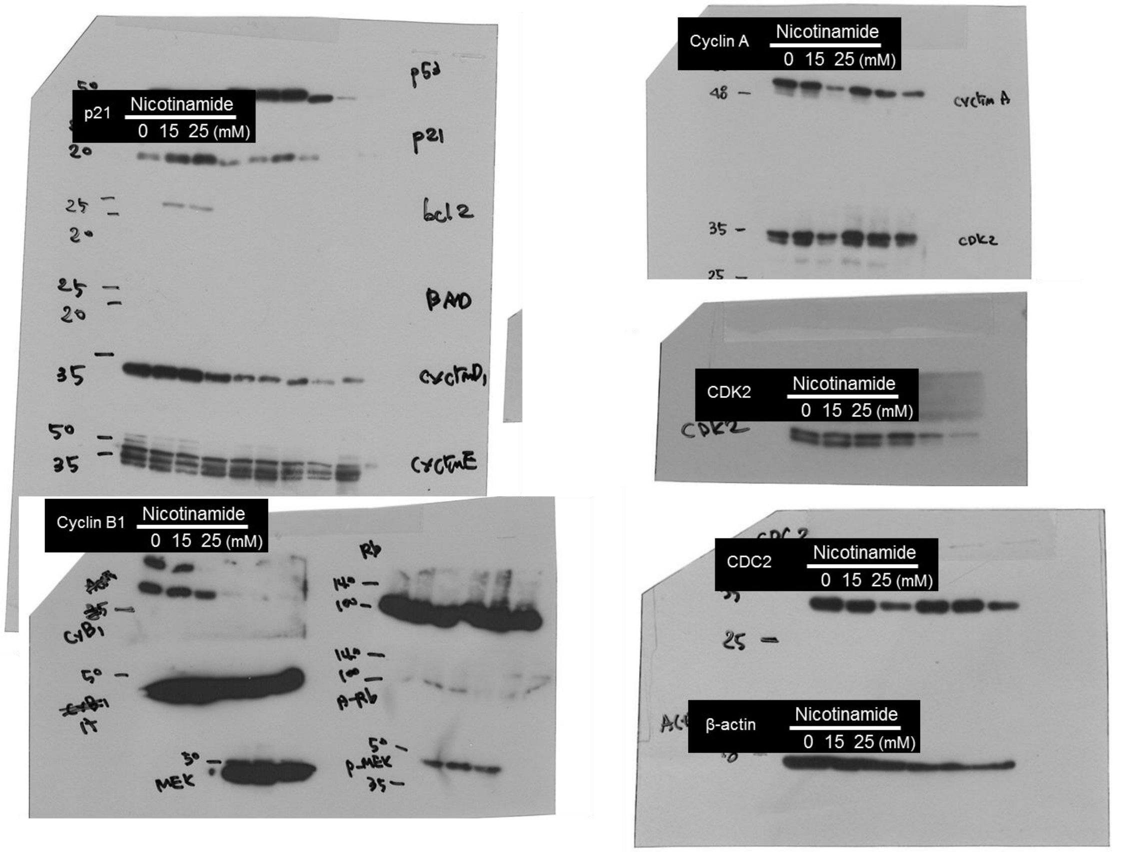

c

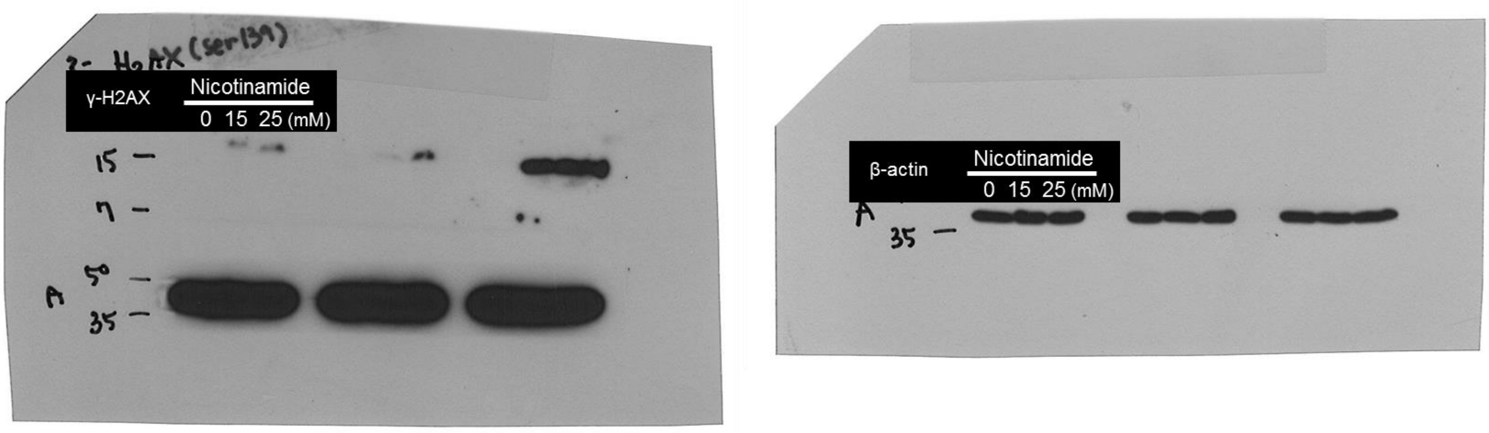
γ-H2AX
Nicotinamide
0 15 25 (mM)
β-actin
Nicotinamide
0 15 25 (mM)

d

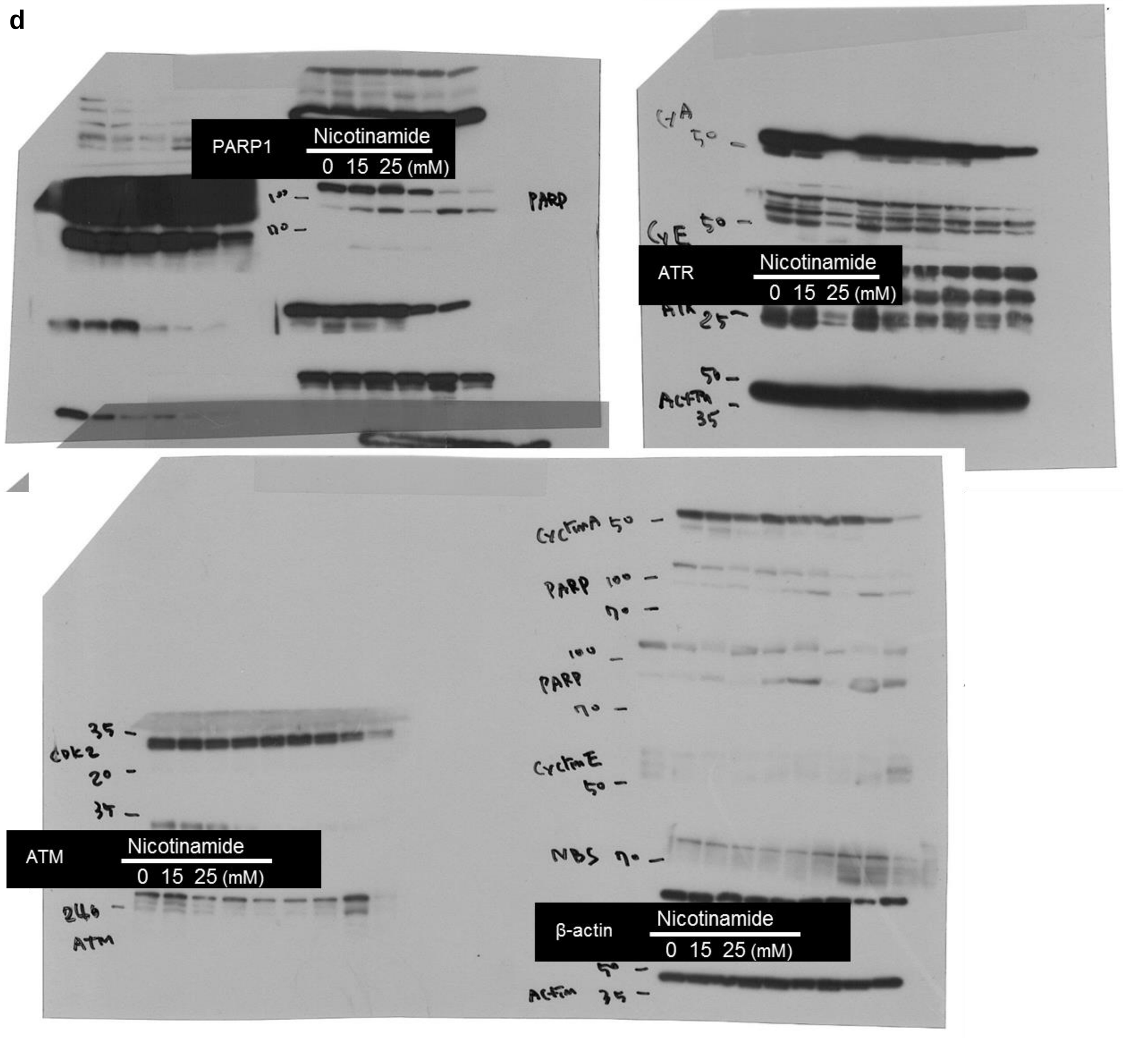
PARP1
Nicotinamide
0 15 25 (mM)
ATR
Nicotinamide
0 15 25 (mM)
ATM
Nicotinamide
0 15 25 (mM)
β-actin
Nicotinamide
0 15 25 (mM)

**e**

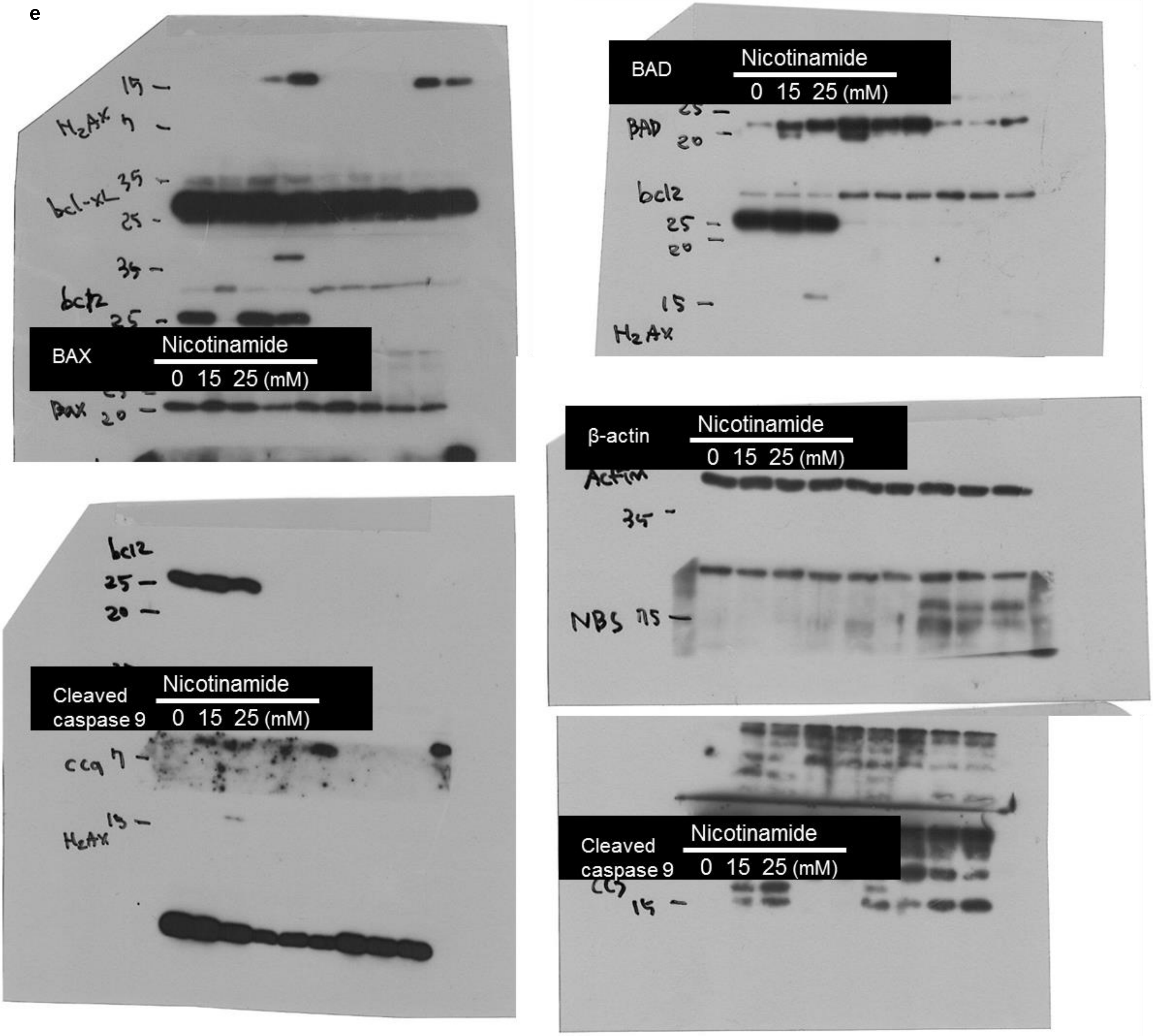
BAX
Nicotinamide
0 15 25 (mM)
BAD
Nicotinamide
0 15 25 (mM)
β-actin
Nicotinamide
0 15 25 (mM)
Cleaved caspase 9
Nicotinamide
0 15 25 (mM)
Cleaved caspase 9
Nicotinamide
0 15 25 (mM)

f

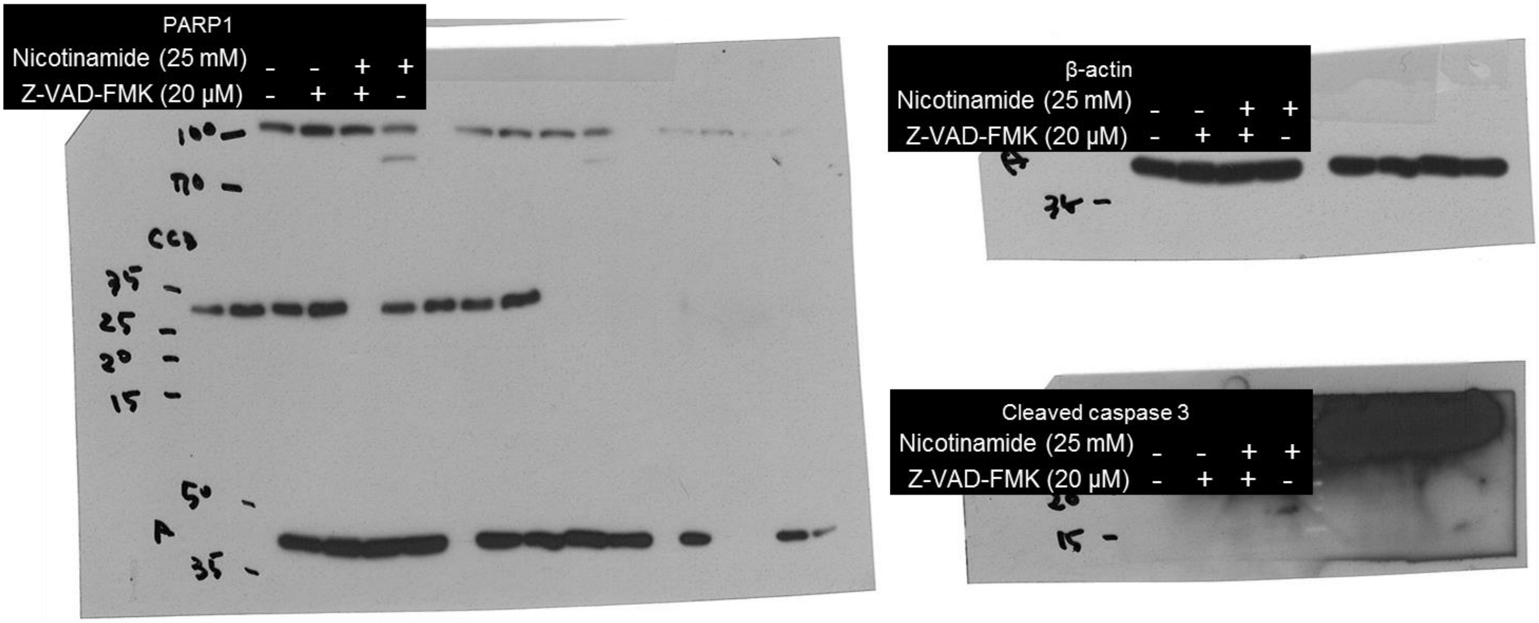
PARP1
Nicotinamide (25 mM) - - + +
Z-VAD-FMK (20 μM) - + + -
β-actin
Nicotinamide (25 mM) - - + +
Z-VAD-FMK (20 μM) - + + -
Cleaved caspase 3
Nicotinamide (25 mM) - - + +
Z-VAD-FMK (20 μM) - + + -

g

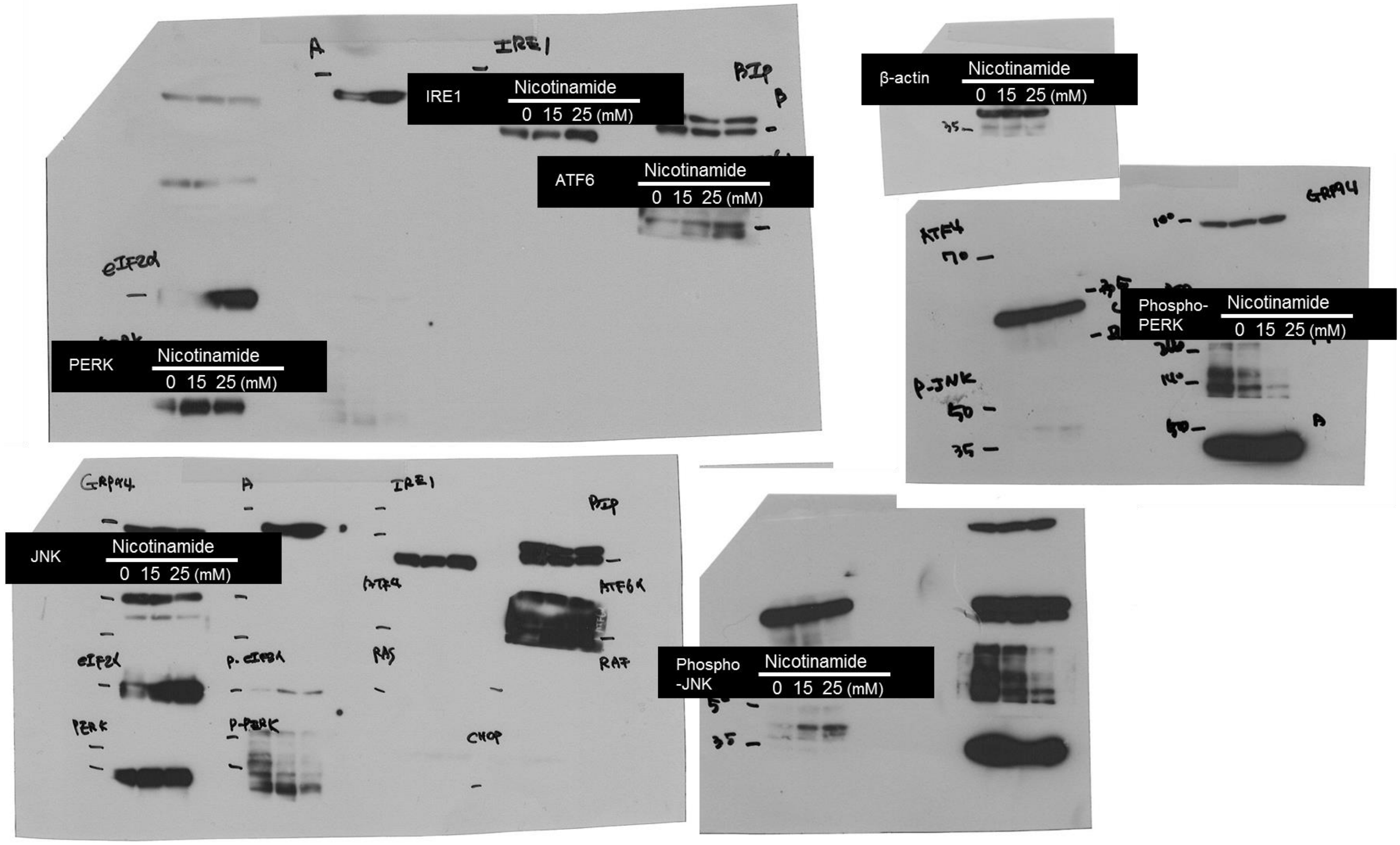
IRE1 Nicotinamide 0 15 25 (mM)
ATF6 Nicotinamide 0 15 25 (mM)
β-actin Nicotinamide 0 15 25 (mM)
PERK Nicotinamide 0 15 25 (mM)
Phospho-PERK Nicotinamide 0 15 25 (mM)
JNK Nicotinamide 0 15 25 (mM)
Phospho -JNK Nicotinamide 0 15 25 (mM)

Supplementary Figure. S13

a

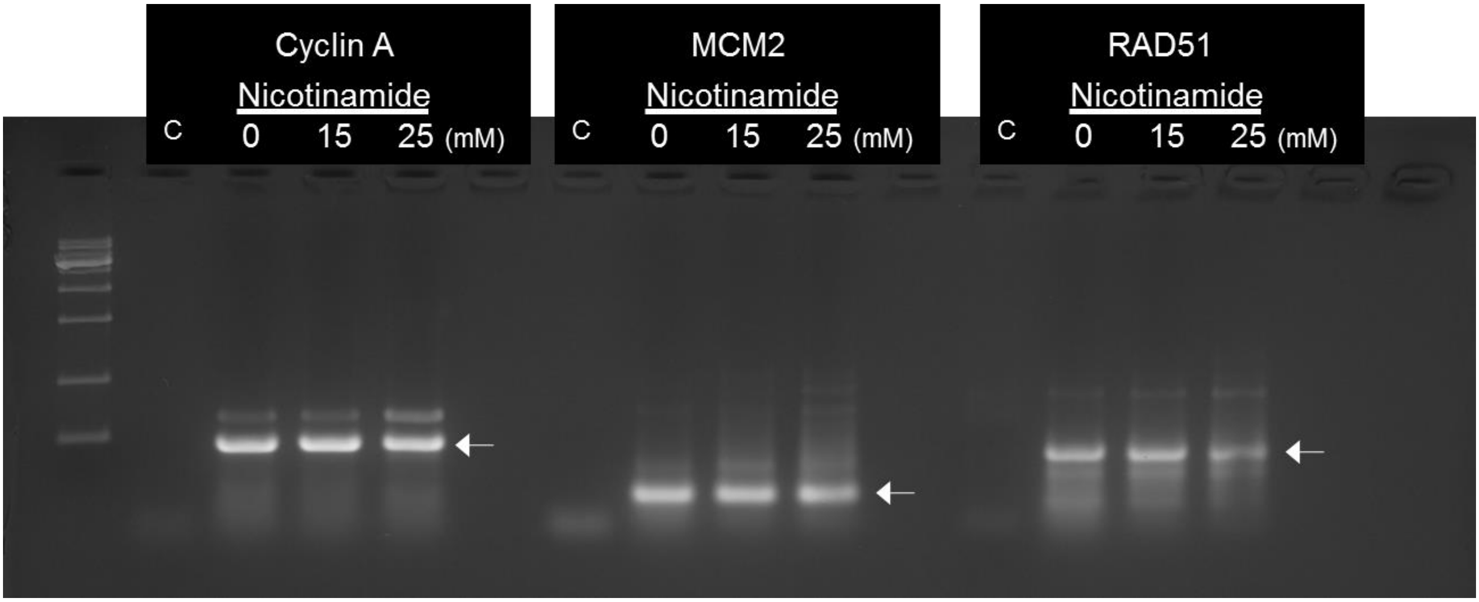


b

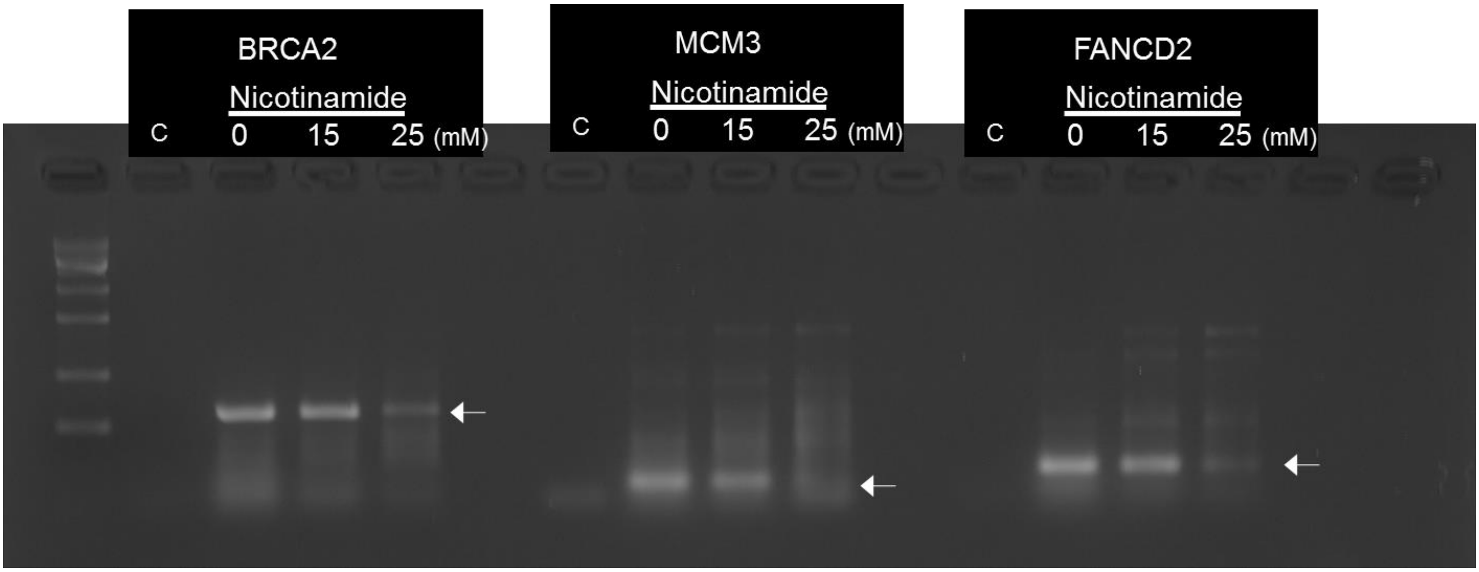

c

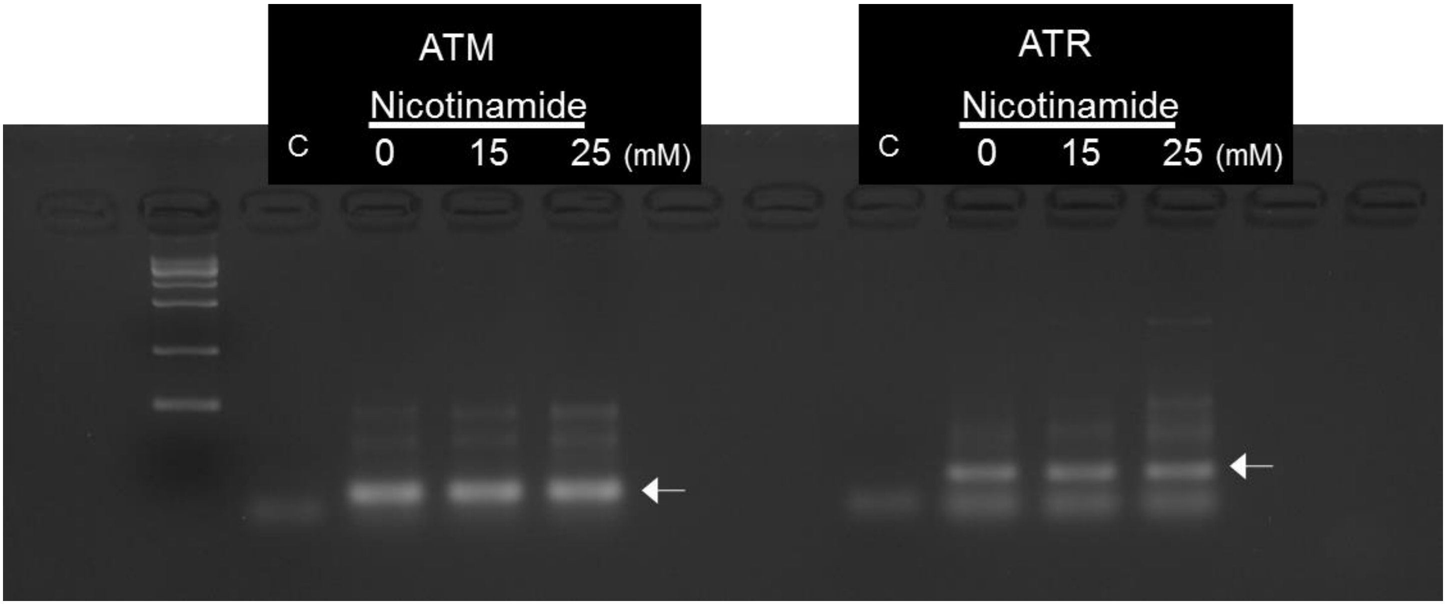

ATM
Nicotinamide
C
0
15
25 (mM)
ATR
Nicotinamide
C
0
15
25 (mM)


d

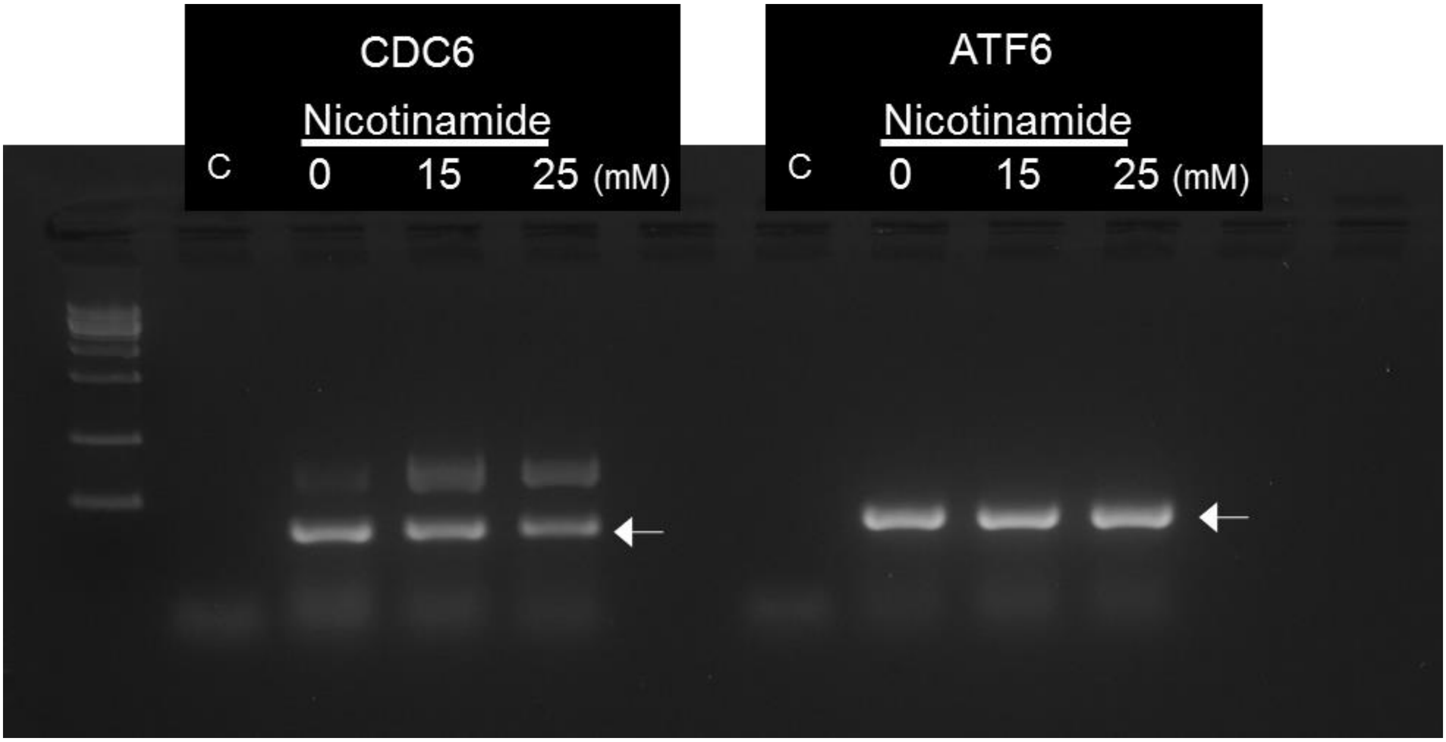

CDC6
Nicotinamide
C
0
15
25 (mM)
ATF6
Nicotinamide
C
0
15
25 (mM)

e

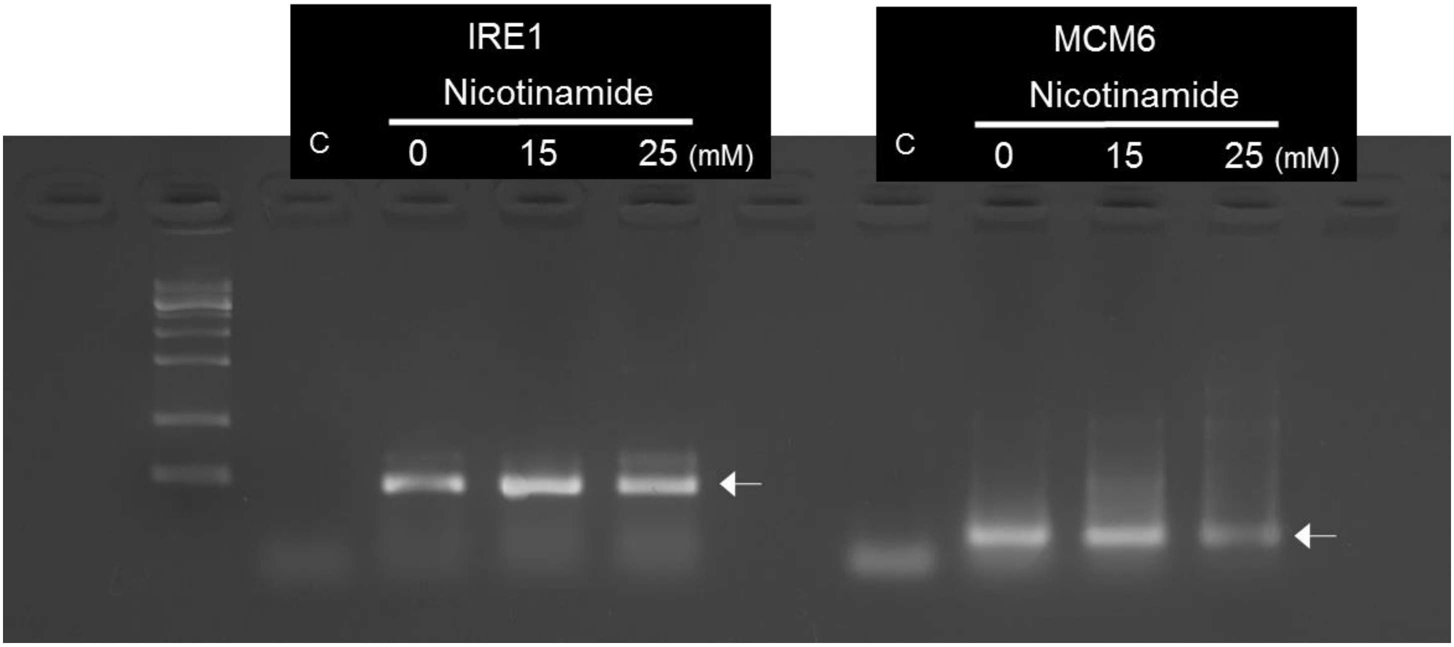
IRE1
Nicotinamide
C
0
15
25 (mM)
MCM6
Nicotinamide
C
0
15
25 (mM)

f

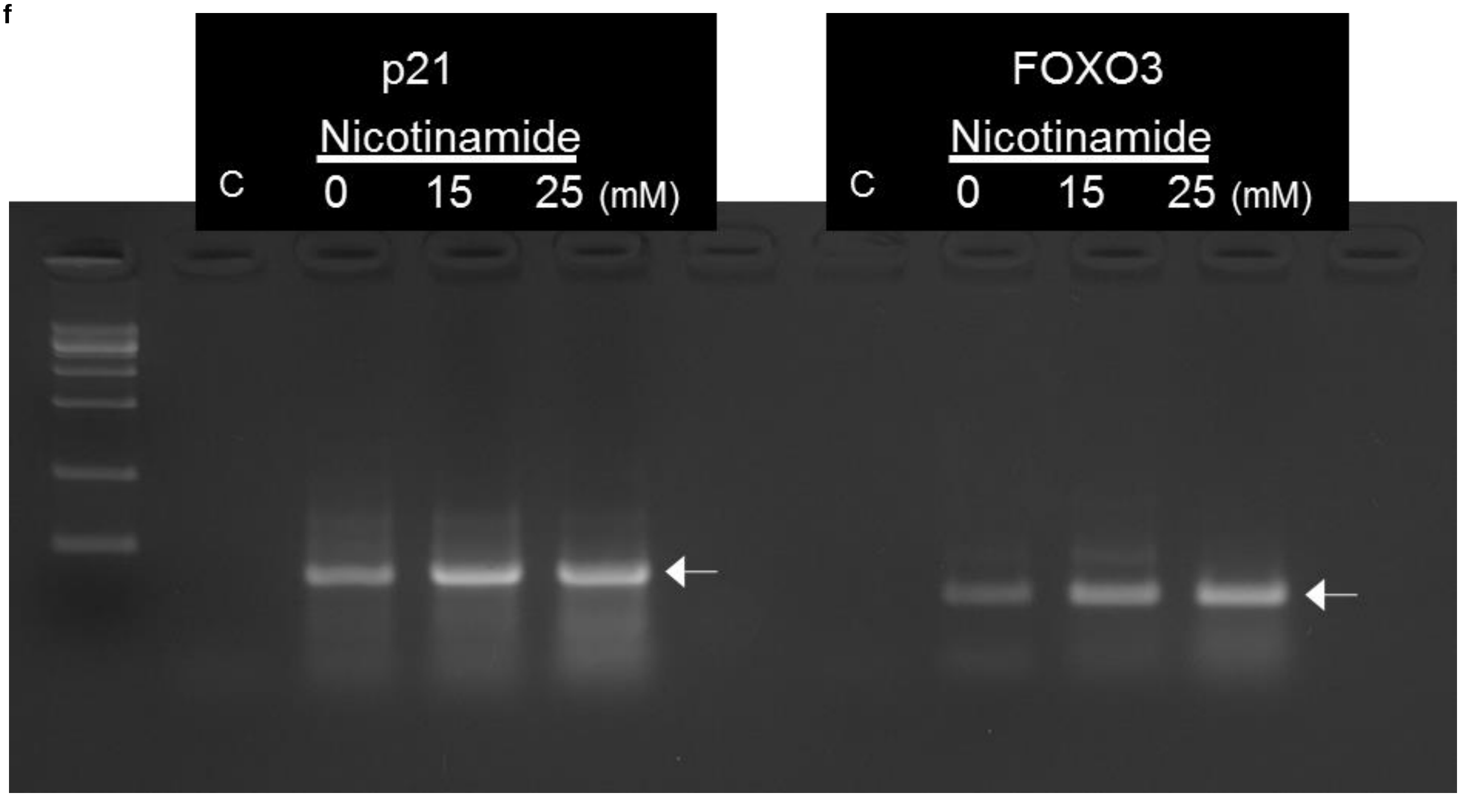
p21
Nicotinamide
C
0
15
25 (mM)
FOXO3
Nicotinamide
C
0
15
25 (mM)

g

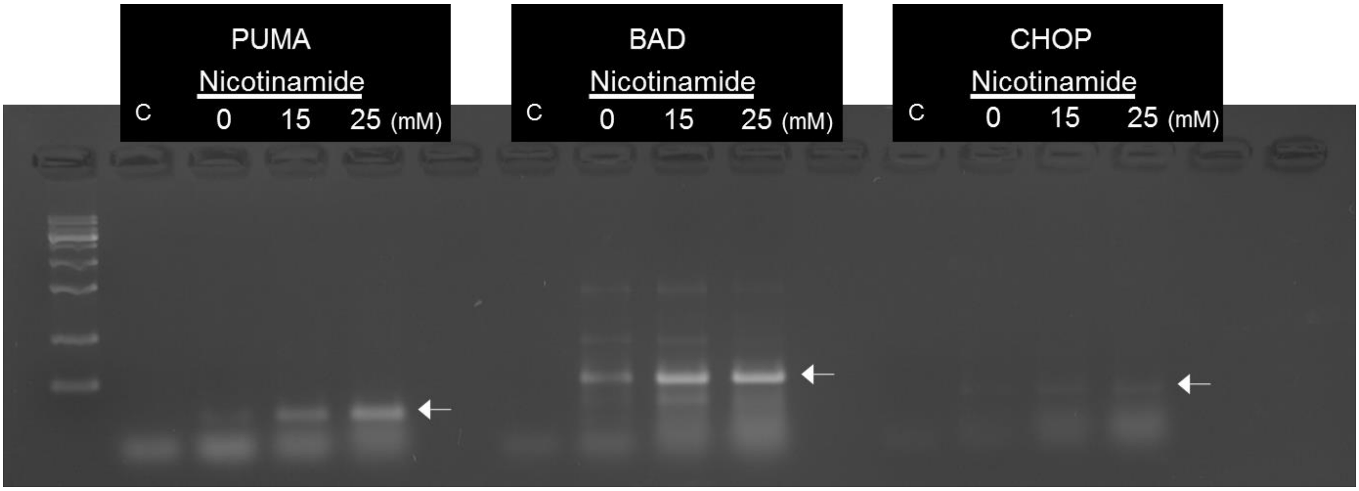
PUMA
Nicotinamide
C 0 15 25 (mM)
BAD
Nicotinamide
C 0 15 25 (mM)
CHOP
Nicotinamide
C 0 15 25 (mM)

h

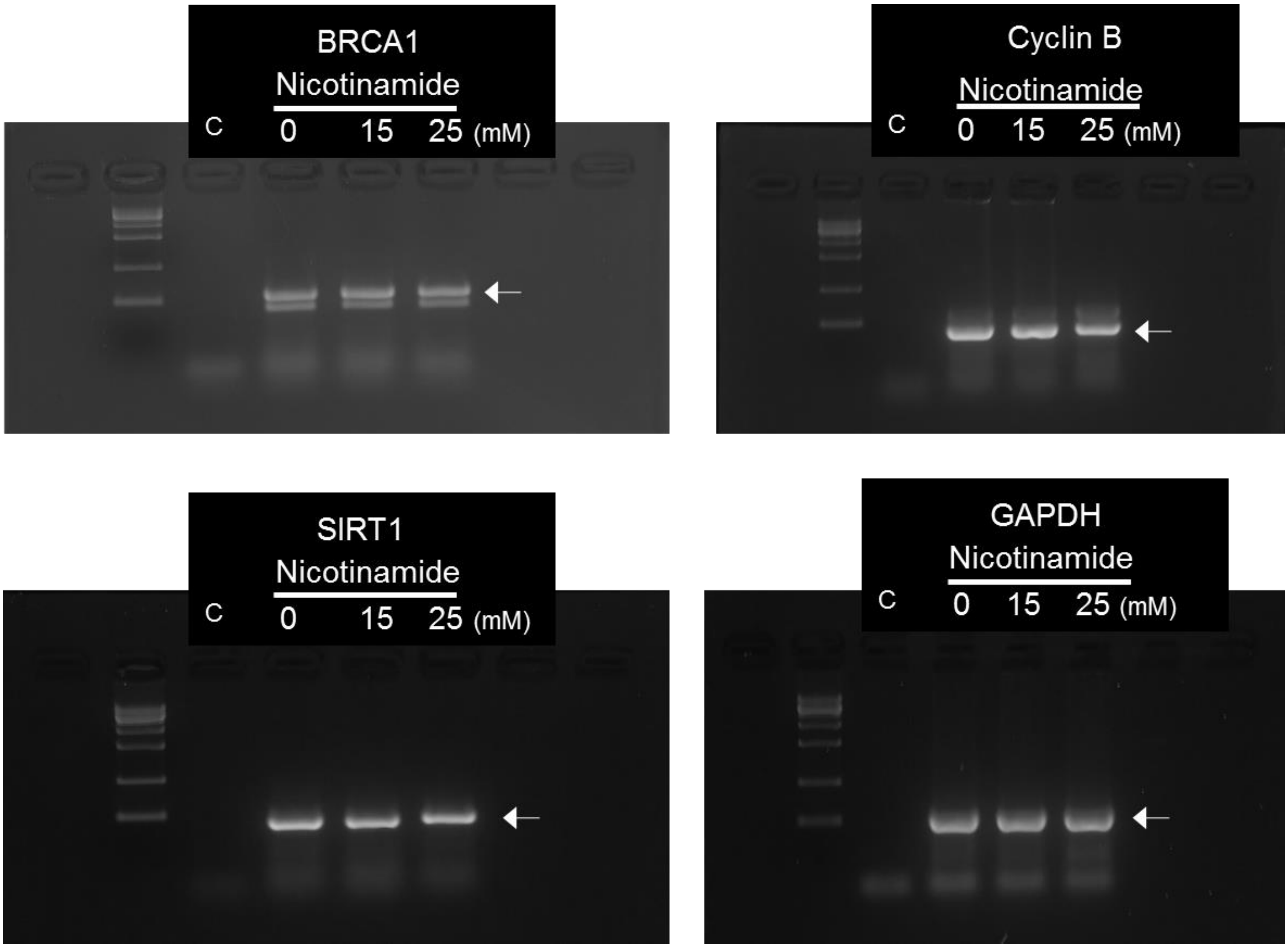
BRCA1
Nicotinamide
C 0 15 25 (mM)
Cyclin B
Nicotinamide
C 0 15 25 (mM)
SIRT1
Nicotinamide
C 0 15 25 (mM)
GAPDH
Nicotinamide
C 0 15 25 (mM)
